# Chemogenetic activation of mammalian brain neurons expressing insect Ionotropic Receptors by systemic ligand precursor administration

**DOI:** 10.1038/s42003-024-06223-4

**Published:** 2024-05-07

**Authors:** Yoshio Iguchi, Ryoji Fukabori, Shigeki Kato, Kazumi Takahashi, Satoshi Eifuku, Yuko Maejima, Kenju Shimomura, Hiroshi Mizuma, Aya Mawatari, Hisashi Doi, Yilong Cui, Hirotaka Onoe, Keigo Hikishima, Makoto Osanai, Takuma Nishijo, Toshihiko Momiyama, Richard Benton, Kazuto Kobayashi

**Affiliations:** 1 Department of Molecular Genetics, Institute of Biomedical Sciences, Fukushima Medical University School of Medicine, 1 Hikarigaoka, Fukushima, 960-1295 Japan; 2 Department of Systems Neuroscience, Fukushima Medical University School of Medicine, 1 Hikarigaoka, Fukushima, 960-1295 Japan; 3 Department of Bioregulation and Pharmacological Medicine, Fukushima Medical University School of Medicine, 1 Hikarigaoka, Fukushima, 960-1295 Japan; 4 Laboratory for Pathophysiological and Health Science, RIKEN Center for Biosystems Dynamics Research, 6-7-3 Minatojima-minamimachi, Chuo-ku, Kobe 650-0047 Japan; 5 Department of Functional Brain Imaging, Institute for Quantum Medical Science, National Institutes for Quantum Science and Technology, 4-9-1 Anagawa, Inage-ku, Chiba 263-8555 Japan; 6 Laboratory for Labeling Chemistry, RIKEN Center for Biosystems Dynamics Research, 6-7-3 Minatojima-minamimachi, Chuo-ku, Kobe 650-0047 Japan; 7 Research, Institute for Drug Discovery Science, Collaborative Creation Research Center, Organization for Research Promotion, Osaka Metropolitan University, 1-1 Gakuen-cho, Naka-ku, Sakai 599-8531 Japan; 8 Laboratory for Biofunction Dynamics Imaging, RIKEN Center for Biosystems Dynamics Research, 6-7-3 Minatojima-minamimachi, Chuo-ku, Kobe 650-0047 Japan; 9 Human Brain Research Center, Kyoto University Graduate School of Medicine, 54 Shogoin-Kawahara-Cho, Sakyo-ku, Kyoto 606-8507 Japan; 10 Medical Devices Research Group, Health and Medical Research Institute, National Institute of Advanced Industrial Science and Technology (AIST), 1-2-1 Namiki, Tsukuba, 305-8564 Japan; 11 Department of Medical Physics and Engineering, Division of Health Sciences, Osaka University Graduate School of Medicine, 1-7 Yamadaoka, Suita, 565-0871 Japan; 12 Department of Pharmacology, Jikei University School of Medicine, 3-25-8 Nishi-shinbashi, Tokyo, 105-8461 Japan; 13 Department of Molecular Neurobiology, Institute for Developmental Research, Aichi Developmental Disability Center, 713-8 Kamiya-cho, Kasugai, 480-0392 Japan; 14 Center for Integrative Genomics, Faculty of Biology and Medicine, University of Lausanne, CH-1015 Lausanne, Switzerland

**Keywords:** Ion channels in the nervous system, Cellular neuroscience, Genetic engineering

## Abstract

Chemogenetic approaches employing ligand-gated ion channels are advantageous regarding manipulation of target neuronal population functions independently of endogenous second messenger pathways. Among them, Ionotropic Receptor (IR)-mediated neuronal activation (IRNA) allows stimulation of mammalian neurons that heterologously express members of the insect chemosensory IR repertoire in response to their cognate ligands. In the original protocol, phenylacetic acid, a ligand of the IR84a/IR8a complex, was locally injected into a brain region due to its low permeability of the blood-brain barrier. To circumvent this invasive injection, we sought to develop a strategy of peripheral administration with a precursor of phenylacetic acid, phenylacetic acid methyl ester, which is efficiently transferred into the brain and converted to the mature ligand by endogenous esterase activities. This strategy was validated by electrophysiological, biochemical, brain-imaging, and behavioral analyses, demonstrating high utility of systemic IRNA technology in the remote activation of target neurons in the brain.

## Abstract (web summary)

This study established a strategy for systemic administration of prodrugs to induce activation in target neurons in the mammalian brain that express Ionotropic Receptors derived from the insect *Drosophila melanogaster*.

## Introduction

Uncovering the roles of specific neuronal populations and neural pathways constituting the complex central nervous system is essential for understanding the mechanisms of higher brain function and the pathophysiology of various psychiatric and neurological disorders. Sophisticated genetic approaches, including transgenic animals and viral vector systems, have provided a powerful experimental strategy for manipulating the functions of these neuronal types and pathways^1,2^. Among them, an increasingly important approach is the chemogenetic manipulation of target neuronal populations^3,4^. A common class of chemogenetic tool is DREADDs, which are genetically engineered G protein-coupled receptors exclusively activated by designer drugs^5,6^. DREADDs have made notable progress in recent years, including a full line-up of engineered receptors^7,8^ and the development of ligands with high affinity to the receptor but minor off-target effects^9,10^. However, they work through altered intracellular signaling of target neurons, which might induce complex cellular reactions^11^. For example, DREADD hM3D (Gq)-dependent pre- and postsynaptic mechanisms exhibited different ligand dose responses^12^. Furthermore, a prolonged high-level DREADD hM4D (Gi) expression resulted in neurotoxic effects^13^.

Another chemogenetic manipulation approach is using ligand-gated ion channels (LGICs) that do not require intracellular signaling pathways to modify neuronal activity^3,14^. LGICs are generally classified into the Cys-loop, P2X, and ionotropic glutamate receptor (iGluR) families. Among them, several approaches depending on Cys-loop receptors have been studied, such as silencing of neuronal activity using glutamate/ivermectin-gated chloride channels derived from *C. elegans*^15,16^, activation of neurons using an ivermectin-sensitive glycine receptor mutant with cation permeability^17^, and a system using chimeric receptors in which the ligand-binding domains of Cys-loop ion channels (pharmacologically selective actuator modules) are activated by cognate synthetic ligands (pharmacologically selective effector molecules)^14,18^. Although an ATP-sensitive P2X2-based tool has been reported in invertebrates^19^, knockout mutant backgrounds are required for application in mammals^3^. It should be noted that not many studies have reported that the activation in the target neuronal population induced by the LGICs-based chemogenetic systems has resulted in the overt behavioral effects^20^.

Recently, we have developed a type of chemogenetic tool, the Ionotropic Receptor (IR)-mediated neuronal activation (IRNA) technology^21^. Insect IRs belong to the iGluR superfamily and are involved in the sensation of a wide diversity of volatile and non-volatile chemicals as well as in the perception of temperature and humidity^22–24^. In particular, we used the heteromer of IR84a and IR8a subunits derived from the insect *Drosophila melanogaster*, which form a cation channel gated by phenylacetic acid (PhAc) and phenylacetaldehyde^25,26^, to induce activation of target neurons in the mammalian brain. The IR84a/IR8a complex was expressed under the control of the promoter for the gene encoding tyrosine hydroxylase (TH) in transgenic (Tg) mice (TH-IR84a/IR8a), and transgene expression was observed in catecholamine-containing neurons, including norepinephrine (NE) neurons in the locus coeruleus (LC)^21^. In a brain slice preparation, the LC-NE neurons in the Tg mice exhibited an increase in membrane potential and firing frequency in response to PhAc and phenylacetaldehyde. Intracranial microinjection of PhAc into the LC activated NE neurons and elevated the extracellular NE level in the terminal regions, resulting in enhancement of the recall process of emotional memory. These findings indicated the efficacy of IRNA technology in activating mammalian target neurons^21^.

In the present study, we developed a strategy of systemic drug administration for the IRNA technology to activate target neurons in the brain. The delivery of IR ligands into the brain by systemic administration is less invasive than intracranial surgery for ligand injection, and permits remote activation of the target neurons with minimal disturbance of the animals, particularly in the case of repeated manipulation^27,28^. Since PhAc is known to have the difficulty in crossing the blood-brain barrier (BBB), we used methyl ester of PhAc (PhAcM) for systemic administration, which exhibits lipophilic properties enhancing BBB permeability by passive diffusion, and is converted to the mature ligand by esterase activities in the brain. We first performed peripheral administration with PhAcM to activate LC-NE neurons in Tg mice. We then applied this strategy for selective activation of striatal projection neurons containing dopamine type 2 receptors in the brain of a Cre transgenic rat line (*Drd2*-Cre), in which IR84a/IR8a transgenes were expressed by using a viral vector carrying the flip-excision switch (FLEX) system. Activation of the target neurons by peripheral administration of the ligand precursors was confirmed by the elevation of GABA release in their terminal regions and the induction of rotational behavior. Furthermore, we visualized the binding of processed ligands in the brain regions expressing the receptor complex using in vivo and ex vivo brain imaging techniques. These results demonstrate that systemic IRNA technology provides a powerful strategy for remote and selective activation of diverse types of target neuronal populations in the mammalian central nervous system.

## Results

### Peripheral administration of methyl ester of PhAc induces excitation of the central IR84a/IR8a-expressing neurons

Transfer of PhAc through the BBB is generally considered to be inefficient^29^. To test whether a peripherally administered PhAc can stimulate the target neurons expressing IR84a and IR8a in the brain, we injected PhAc solution intravenously into the lateral tail vein of the TH-IR84a/IR8a mice and monitored NE release in the anterior cingulate cortex (ACC), a brain region innervated by the LC, by using a microdialysis procedure. When we first tested the peripheral administration of PhAc (10 or 30 mg/kg), the extracellular NE level (NE_[ext]_) in the ACC did not show any significant changes as compared to vehicle administration (Supplementary Fig. 1a). In contrast, perfusion of different concentrations of PhAc into the dialysis probe (i.e., a reverse dialysis with PhAc) elevated NE_[ext]_ in a dose-dependent manner in Tg animals but not in non-Tg animals (Supplementary Fig. 1b). These data indicate that the central IR84a/IR8a-expressing neurons are activated by intracranially administered PhAc, but have a low response to peripherally administered PhAc.

**Fig. 1:**
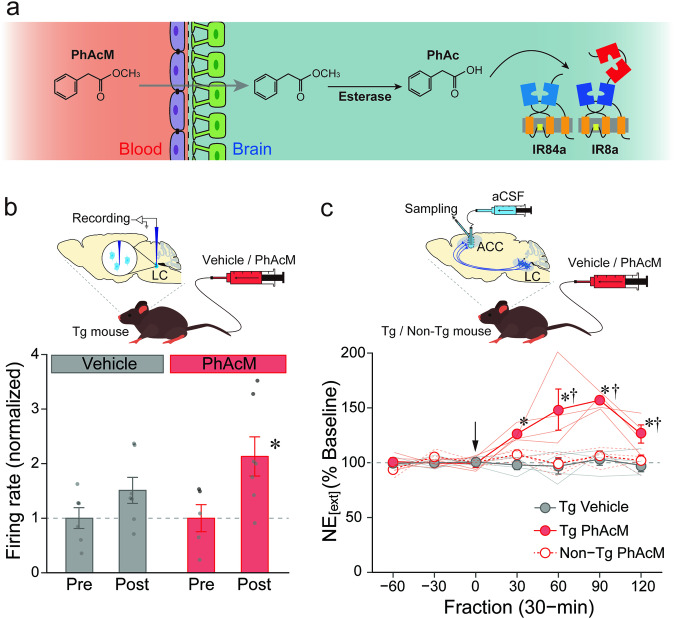
Enhancement of the BBB permeability of PhAc by methyl esterification. **a** Schematic representation of drug delivery. PhAcM in the circulating blood is translocated across the BBB and enters the brain, where PhAcM is converted to PhAc by endogenous esterase activity and PhAc activates the IR84a/IR8a complex. **b** Plot of changes in the normalized firing rate after the tail vein injection of PhAcM (20 mg/kg) or vehicle in the TH-IR84a/IR8a (Tg) mice (*n* = 6 mice for vehicle/pre, *n* = 7 mice for vehicle/post, *n* = 5 mice for PhAcM/pre, and *n* = 7 mice for PhAcM/post). ^*^p < 0.05 vs. PhAcM/pre. **c** Plot of changes in NE_[ext]_ in the ACC of Tg and non-transgenic (non-Tg) littermates before and after lateral tail vein injection of PhAcM (10 mg/kg) (*n* = 4 mice for each group). NE_[ext]_ is expressed as a percentage of the average baseline levels of each mouse. Arrow indicates the timing of drug injection. NE_[ext]_ in PhAcM-administered Tg mice was significantly higher than that of the vehicle-administered Tg mice at the 30- to 120-min fractions (*t*s_(63)_ = 3.38, 6.12, 6.43, 3.41, *p* = 0.004, *p* < 0.001, *p* < 0.001, *p* = 0.003, *r*s = 0.39, 0.61, 0.63, 0.39, respectively) and that of the PhAcM-administered non-Tg at the 60- to 120-min fractions (*t*s_(63)_ = 5.90, 6.06, 2.89, *p* < 0.001, *p* < 0.001, *p* = 0.016, *r*s = 0.60, 0.61, 0.34, respectively). ^*^p < 0.05 vs. Tg Vehicle; ^†^*p* < 0.05 vs. Non-Tg PhAcM. Data are presented as mean with accompanying error bar (standard error of the mean or SEM) and individual data points superimposed (**b, c**).

Methyl ester derivatives of carboxylic acid possess a higher BBB permeability and the derivatives are converted to the carboxylic acid form by esterase activities in the brain^30–32^. Thus, we investigated whether phenylacetic acid methyl ester (PhAcM) works as a ligand precursor that is efficiently delivered into the brain through the BBB and converted to PhAc by esterase activity, resulting in the activation of target neurons (see Fig. 1a for a model of drug delivery). We administered a PhAcM solution (20 mg/kg) or vehicle intravenously into the lateral tail vein of the Tg mice, and monitored the firing rate of the LC neurons using an extracellular recording. The normalized firing rate was significantly elevated following PhAcM administration (Fig. 1b; post vs. pre, one-way ANOVA, F_1, 10_ = 5.62, *p* = 0.039, partial *η*^2^ = 0.36), but not following vehicle administration (F_1, 11_ = 2.63, *p* = 0.133, partial *η*^2^ = 0.19).

Next, we examined changes in NE_[ext]_ in the ACC of the Tg mice by the peripheral PhAcM administration. PhAcM administration resulted in a marked increase in NE_[ext]_ in the Tg mice (Fig. 1c; two-way ANOVA, group effect: F_2, 9_ = 24.95, *p* < 0.001, partial *η*^2^ = 0.84, fraction effect: F_6, 54_ = 6.81, *p* < 0.001, partial *η*^2^ = 0.43, interaction: F_12, 54_ = 5.58, *p* < 0.001, partial *η*^2^ = 0.55). These data show that peripheral administration of methyl ester of PhAc efficiently stimulates the IR84a/IR8a-expressing target neurons in the brain.

### Processing of the ligand precursor results in the activation of the IR84a/IR8a-expressing neurons in the brain

**Fig. 2:**
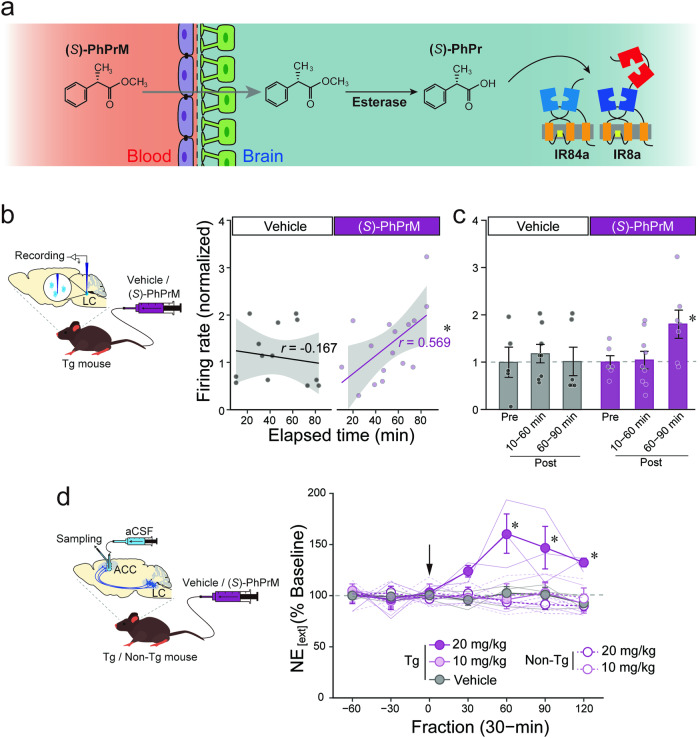
Identification of (*S*)-PhPr as an IR84a/IR8a ligand and enhancement of the BBB permeability of (*S*)-PhPr by methyl esterification. **a** Schematic representation of enhanced BBB permeability of PhPr. It was expected that peripherally administered methyl ester of PhPr (PhPrM) penetrates the brain across the BBB and converted PhPr stimulates the IR84a/IR8a complex. **b** Plot of correlation between the normalized firing rate of the LC neurons and the elapsed time (min) after (*S*)-PhPrM (20 mg/kg) or vehicle tail vein injection in the Tg mice (vehicle *n* = 14 neurons, (*S*)-PhPrM *n* = 16 neurons). ^*^p < 0.05. **c** Plot of changes in the normalized firing rate by the (*S*)-PhPrM (20 mg/kg) and vehicle tail vein injection in the Tg mice (vehicle/pre *n* = 5 neurons, vehicle/post 10–60 min *n* = 8 neurons, vehicle/post 60–90 min *n* = 6 neurons, (*S*)-PhPrM/pre *n* = 6 neurons, (*S*)-PhPrM/post 10–60 min *n* = 9 neurons, (*S*)-PhPrM/post 60–90 min *n* = 7 neurons). ^*^p < 0.05 vs. (*S*)-PhPrM/pre. **d** Plot of changes in the extracellular NE level (NE_[ext]_) in the ACC of the Tg and non-Tg mice before and after (*S*)-PhPrM (10 or 20 mg/kg) tail vein injection (*n* = 3 mice for all groups). NE_[ext]_ is expressed as a percentage of the average baseline levels of each mouse. Arrow indicates the timing of drug injection. At the 60- to 120-min fractions, NE_[ext]_ of 20 mg/kg (*S*)-PhPrM -administered Tg mice was significantly higher than that of vehicle- (*t*s_(70*)*_ = 5.18, 4.08, 3.55, *p*s < 0.001, *r*s = 0.51, 0.44, 0.39, respectively) or 10 mg/kg (*S*)-PhPrM-administered Tg mice (*t*s_(70)_ = 5.75, 3.93, 3.57, *p*s < 0.001, *p* = 0.001, *r*s = 0.57, 0.43, 0.39, respectively) and that of 10 mg/kg (*t*s_(70)_ = 5.36, 3.90, 3.07, *p*s < 0.001, *p* = 0.003, *r*s = 0.54, 0.42, 0.34, respectively) or 20 mg/kg (*S*)-PhPrM-administered non-Tg littermates (*t*s_(70)_ = 5.94, 4.96, 3.77, *p*s < 0.001, *r*s = 0.58, 0.51, 0.41, respectively).^*^p < 0.05 vs. other four groups. Data are presented as mean with accompanying error bar (SEM) and individual data points superimposed (**c, d**).

Our in vivo electrophysiological and biochemical experiments demonstrated that peripherally administered PhAcM induces excitation of the central IR84a/IR8a-expressing neurons (Fig. 1b, c), suggesting that a precursor of the ligand is converted to the ligand by endogenous esterase following translocation across the BBB. To test this possibility, we synthesized a ^11^C-incorporated analog of PhAcM, 2-phenyl[3-^11^C]propionic acid methyl ester ([^11^C]PhPrM), in which the methyl group at the 3-position of 2-phenylpropionic acid methyl ester (PhPrM) was labeled with ^11^C. Although there were two stereoisomers with the (*S*)- and (*R*)-configurations regarding 2-phenylpropionic acid (PhPr), we demonstrated an increase in activity of the IR84a/IR8a-expressing neurons only by (*S*)-PhPr using an ex vivo whole-cell current clamp recording and in vivo microdialysis with the LC-NE system of the Tg mice (Supplementary Fig. 3), as well as a dose-dependent current response to (*S*)-PhPr using a whole-cell voltage clamp recording with the cultured cells (Supplementary Fig. 4). Thus, we modeled that peripherally administered methyl ester form (*S*)-PhPrM crosses the BBB and enters the brain, where (*S*)-PhPrM is metabolized to its acid form (*S*)-PhPr by esterase activity (Fig. 2a). We injected (*S*)-[^11^C]PhPrM into the lateral tail vein of the Tg mice and removed their brains 30 min later. Tissue suspension was prepared from the LC region and subjected to thin-layer chromatography (TLC) analysis (Supplementary Fig. 5). The results indicate the presence of (*S*)-[^11^C]PhPr in the LC tissue, supporting the model for the translocation of peripherally administered (*S*)-[^11^C]PhPrM across the BBB into the brain, and the following conversion to (*S*)-[^11^C]PhPr by esterase activity.

We next tested whether peripherally administered (*S*)-PhPrM stimulates central noradrenergic activity in Tg mice using in vivo extracellular recording. The normalized firing rate of the LC-NE neurons showed a gradual increase following injection of (*S*)-PhPrM (20 mg/kg) into the lateral tail vein of the Tg mice, whereas no such increase was observed following vehicle injection (Fig. 2b); statistical tests supported the observation, and Pearson correlation (*r*) between the elapsed time after drug administration and firing rate (normalized) was significant for (*S*)-PhPrM (*r* = 0.569, *p* = 0.021) but not for vehicle (*r* = -0.167, *p* = 0.567). When we divided the data into three time periods (pre-administration, 10–60 min after administration, and 60–90 min the administration) and compared the mean firing rate for each period (Fig. 2c), the mean firing rate up to 60 min after (*S*)-PhPrM administration (10–60 min) was not significantly different from that of pre-administration (one-way ANOVA, F_1, 13_ = 0.03, *p* = 0.872, partial *η*^2^ = 0.002), but the mean firing rate after 60 min of (*S*)-PhPrM administration (60–90 min) was significantly higher than that of pre-administration (F_1, 11_ = 5.38, *p* = 0.041, partial *η*^2^ = 0.33). In the vehicle condition, there were no significant differences between pre and 10–60-min post-administration (F_1, 11_ = 0.27, *p* = 0.614, partial *η*^2^ = 0.02) or pre and 60–90-min post-administration (F_1, 9_ = 0.001, *p* = 0.970, partial *η*^2^ = 0.0001).

Furthermore, we examined the change in NE_[ext]_ in the ACC of the Tg and non-Tg mice by the peripheral (*S*)-PhPrM administration (Fig. 2d). A 20 mg/kg (*S*)-PhPrM resulted in a slightly delayed but long-lasting increase in NE_[ext]_ in the Tg mice (two-way ANOVA, group effect: F_4, 10_ = 9.73, *p* = 0.002, partial *η*^2^ = 0.80, fraction effect: F_6, 24_ = 1.93, *p* = 0.090, partial *η*^2^ = 0.16, interaction: F_6, 60_ = 2.45, *p* = 0.003, partial *η*^2^ = 0.49). These data show that the peripherally administered ligand precursor (*S*)-PhPrM is converted to (*S*)-PhPr in the brain following translocation across the BBB, which stimulates the IR84a/IR8a-expressing LC-NE neurons.

### Functional expression of IR84a/IR8a in specific cell types by using a viral vector system

**Fig. 3:**
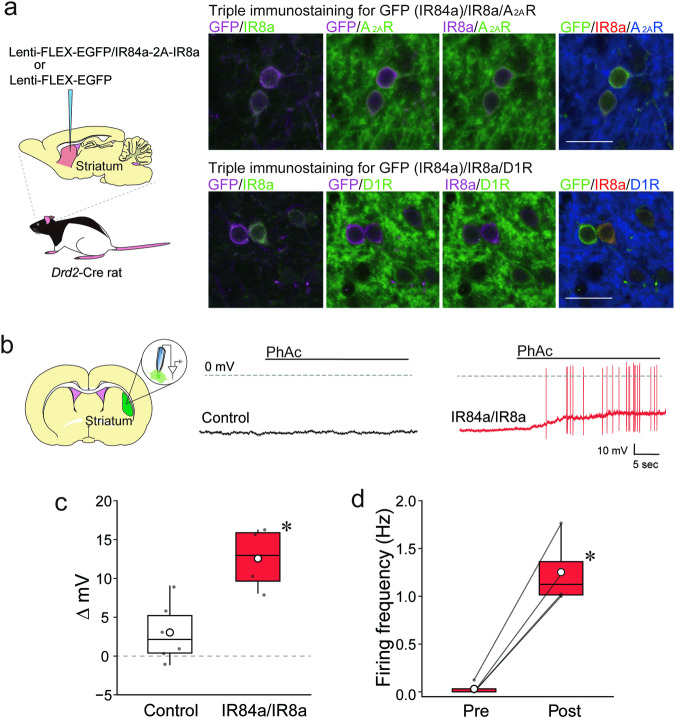
Cell type-specific expression of IR84a/IR8a in the rat striatum and responses to PhAc. **a** Robust expression of IR84a and IR8a in iSPNs (A_2A_R + ), but not in dSPNs (D1R + ) in Tg rats. A lentiviral vector expressing EGFP/IR84a and IR8a in a Cre-dependent manner was microinjected into the striatum of the *Drd2*-Cre rats. Sections were triple-stained through immunohistochemistry for GFP, IR8a, and A_2A_R, or GFP, IR8a, and D1R. Scale bar: 25 μm. **b** Representative whole-cell recordings of the Lenti-FLEX-EGFP (control) or Lenti-FLEX-EGFP/IR84a-2A-IR8a (IR84a/IR8a) vector-treated striatal neurons of the *Drd2*-Cre rats in response to PhAc. **c, d** Plot of changes in the membrane potential (**c**) and firing frequency (**d**) with addition of PhAc (7.3 mM, 0.1% w/v) in the control (*n* = 6 neurons) and IR84a/IR8a-expressing neurons (*n* = 4 neurons). ^*^p < 0.05. Data are presented as boxplots with mean values represented by white circles and individual data points superimposed.

We examined whether the IRNA technology could be applied to non-NE neurons in species other than mice using a viral vector strategy carrying the FLEX system^33–35^. For this aim, we employed the striatum of rats as a model. The striatum controls behaviors through the activity distributed across two subpopulations of GABAergic spiny projection neurons (SPNs), which express distinct dopamine receptor subtypes that respond to dopamine in an opposing manner^36,37^: direct SPNs (dSPNs) express type 1 receptors (D1Rs) and project to the substantia nigra pars reticulata and entopeduncular nucleus; indirect SPN (iSPNs) express type 2 receptors (D2Rs), together with adenosine A_2A_ receptor (A_2A_R)^38,39^, sending axons to the external segment of the globus pallidus (GPe). We employed *Drd2*-Cre transgenic rats that express Cre recombinase predominantly in iSPNs^40^. Triple immunohistochemistry of the section through the striatum of the *Drd2*-Cre rats for Cre, A_2A_R, and D1R established that the Cre transgene was highly and specifically expressed in iSPNs but was absent in dSPNs (Supplementary Fig. 7). A lentiviral vector pseudotyped with vesicular stomatitis virus glycoprotein (VSV-G) was constructed to express EGFP/IR84a-2A-IR8a in a Cre-dependent manner (Lenti-FLEX-EGFP/IR84a-2A-IR8a) and then microinjected into the dorsal striatum of the *Drd2*-Cre rats (Fig. 3a). Striatal sections were stained by triple immunohistochemistry for GFP, IR8a, and A_2A_R, or GFP, IR8a, and D1R (Fig. 3a). The numbers of immuno-positive cells (total GFP^+^, total IR8a^+^, GFP^+^ & IR8a^+^, total A_2A_R^+^, total D1R^+^, GFP^+^ & A_2A_R^+^, GFP^+^ & D1R + , IR8a^+^ & A_2A_R^+^, IR8a^+^ & D1R^+^, GFP^+^ & IR8a^+^ & A_2A_R^+^, GFP^+^ & IR8a^+^ & D1R^+^) were counted (Supplementary Table 1). The frequencies of EGFP/IR84a and IR8a double-expression in two types of neurons (efficiency) were 73.92 ± 0.01% in the A_2A_R-positive neurons and 2.52 ± 0.01% in the D1R-positive neurons (*n* = 4 animals), and the frequencies of two types of neurons in EGFP/IR84a and IR8a double-expressing neurons (specificity) were 79.87 ± 0.03% for the A_2A_R-positive neurons and 2.28 ± 0.01% for the D1R-positive neurons (*n* = 4 animals), indicating the efficient and specific expression of EGFP/IR84a and IR8a in striatal iSPNs in the viral vector-injected *Drd2*-Cre rats.

To determine whether striatal cells expressing IR84a/IR8a show neuronal activation by PhAc, we induced expression of either both EGFP/IR84a and IR8a or GFP alone (as the control condition) in striatal iSPNs using the lentiviral vector system and performed a whole-cell current clamp recording in slice preparations (Fig. 3b). The difference in the membrane potential between pre- and post-PhAc (7.3 mM, 0.1% w/v) bath application (Δ mV) of striatal neurons expressing IR84a and IR8a was significantly greater than that of the control neurons expressing GFP but not IR84a/IR8a (Fig. 3c; one-way ANOVA, genotype effect: F_1, 8_ = 14.03, *p* = 0.006, partial *η*^2^ = 0.64). The IR84a/IR8a-expressing neurons also showed significant elevation in the firing frequency by PhAc application (Fig. 3d; one-way ANOVA, genotype effect: F_1, 3_ = 69.61, *p* = 0.004, partial *η*^2^ = 0.96). These data suggest that the viral vector gene expression system allows a cell type-specific, highly efficient expression of IR84a/IR8a, and that cells expressing the two subunits show neuronal activation in a ligand-dependent manner.

### Peripheral administration of the ligand precursors induces excitation of target neurons expressing IR84/IR8a with a viral vector system

**Fig. 4:**
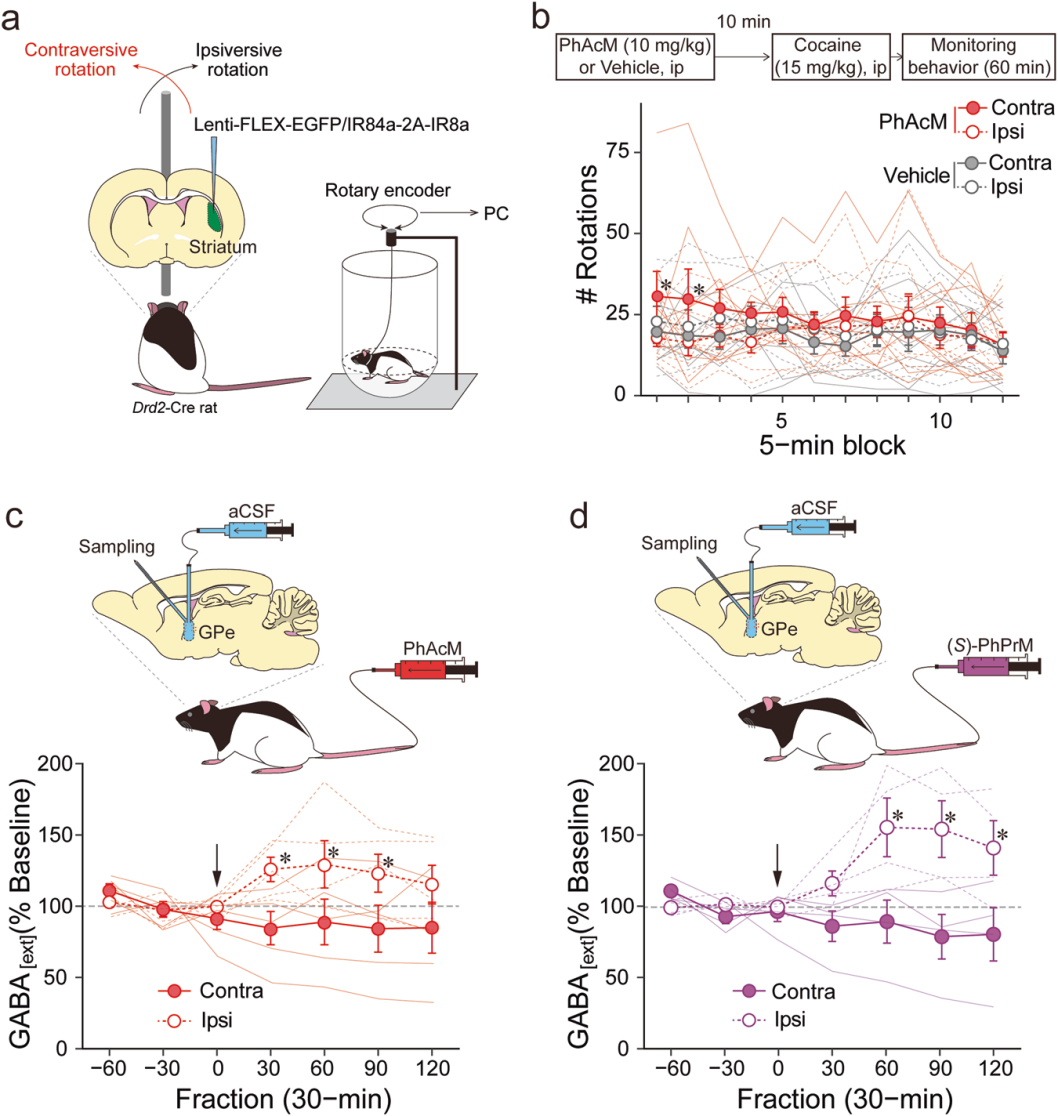
Effects of peripherally administered ligand-dependent activation of the IR84a/IR8a expressing iSPNs. **a** Schematic representation of behavioral paradigm to monitor PhAcM-dependent motor effects. The *Drd2*-Cre rats received the lentiviral vector that induces IR84a/IR8a in a Cre-dependent manner in the unilateral striatum. Their rotational behavior was monitored in a cylinder with hemispheric bottom via a rotary encoder connected to PC. **b** Schedule of the rotational behavior test and plot of changes in the rats’ contraversive and ipsiversive rotations induced by cocaine following PhAcM injection. Rats were intraperitoneally (ip) injected with PhAcM (10 mg/kg) or vehicle, and then cocaine ip after a 10-min interval, followed by behavioral monitoring in the apparatus (*n*s = 8 rats for vehicle and PhAcM conditions). ^*^p < 0.05 vs. PhAcM/ipsi. **c** Plot of changes in the extracellular GABA level (GABA_[ext]_) in the GPe of the viral vector-treated *Drd2*-Cre rats before and after PhAcM (10 mg/kg) tail vein injection. GABA_[ext]_ is expressed as a percentage of the average baseline levels of each rat. Arrows indicate the timing of drug injection. Ipsilateral and contralateral GPes of the viral vector-treated striatum were compared (*n* = 5 rats). GABA_[ext]_ in the ipsilateral GPe was significantly higher than that of the contralateral GPe at the 30- to 90-min fractions (*F*s_(1, 56)_ = 6.42, 6.19, 5.73, *p*s = 0.014, 0.016, 0.020, partial *η*^2^ = 0.014, 0.016, 0.020, respectively). ^*^p < 0.05. **d** Plot of changes in GABA_[ext]_ in the GPe of the viral vector-treated *Drd2*-Cre rats before and after PhPrM (20 mg/kg) tail vein injection. Ipsilateral and contralateral GPes of the viral vector-treated striatum were compared (*n* = 4 rats). GABA_[ext]_ in the ipsilateral GPe was significantly higher than that in the contralateral GPe at the 60- to 120-min fractions (*F*s_(1, 42)_ = 13.32, 17.34, 11.19, *p* = 0.001, *p* < 0.001, *p* = 0.002, partial *η*^2^ = 0.37, 0.43, 0.33, respectively). ^*^p < 0.05. Data are presented as mean with accompanying error bar (SEM) and individual data points superimposed (**b, c, d**).

Striatal neurons of rats expressing IR84a/IR8a by the viral vector system were responsive to PhAc in the slice preparation. We then investigated whether these neurons respond to peripherally administered ligand precursors in vivo. We expressed EGFP/IR84a and IR8a in the iSPNs in the unilateral striatum (left or right) of the *Drd2-Cre* rats by microinjection of the lentiviral vector, then examined whether cocaine-induced dopaminergic activation of the striatum is modulated by the peripherally administered PhAcM, resulting in a rotating motion to a specific direction (Fig. 4a). Rats were injected intraperitoneally with cocaine (15 mg/kg), and their behavior was monitored 10 min after intraperitoneal administration of PhAcM (10 mg/kg), since our in vivo electrophysiological experiments with Tg mice showed that peripheral PhAcM administration resulted in significant elevation of the target neurons’ firing rate after 10 min (Fig. 1b). Contraversive rotation was observed in the rats when PhAcM was administered but no such directional bias occurred when the vehicle was administered (Fig. 4b; three-way ANOVA, drug effect: F_1, 7_ = 0.45, *p* = 0.542, partial *η*^2^ = 0.06, direction effect: F_1, 7_ = 0.09, *p* = 0.780, partial *η*^2^ = 0.01, time-bin effect: F_11, 77_ = 1.48, *p* = 0.156, partial *η*^2^ = 0.17, drug × direction interaction: F_1, 7_ = 9.49, *p* = 0.018, partial *η*^2^ = 0.58, drug × time-bin interaction: F_11, 77_ = 0.33, *p* = 0.977, partial *η*^2^ = 0.04, direction × time-bin interaction: F_11, 77_ = 0.82, *p* = 0.620, partial *η*^2^ = 0.10, drug × direction × time-bin interaction: F_11, 77_ = 2.22, *p* = 0.022, partial *η*^2^ = 0.24); the number of contraversive rotations after administration of PhAcM was significantly higher than that after administration of the vehicle in the first and second 5-min bin (Fs_1, 168_ = 5.04, 5.44, *p*s = 0.026, 0.021, partial *η*^2^s = 0.21, 0.23).

We then examined changes in GABA release in the GPe via intravenous administration of (*S*)-PhPrM (20 mg/kg) as well as PhAcM (10 mg/kg), with the rats completing the behavioral experiment. The effects of these drugs on extracellular GABA level (GABA_[ext]_) were assessed in the ipsilateral GPe to the viral vector-injected striatum with the contralateral GPe (ipsilateral to the intact striatum) as the control condition. PhAcM administration resulted in a rapid and sustained increase in GABA_[ext]_ in the ipsilateral GPe relative to the contralateral GPe (Fig. 4c; two-way ANOVA, hemisphere effect: F_1, 8_ = 3.76, *p* = 0.089, partial *η*^2^ = 0.32, fraction effect: F_6, 48_ = 0.61, *p* = 0.719, partial *η*^2^ = 0.07, interaction: F_6, 48_ = 2.69, *p* = 0.025, partial *η*^2^ = 0.25). (*S*)-PhPrM resulted in a slightly delayed but long-lasting increase in GABA_[ext]_ in the ipsilateral GPe relative to the contralateral GPe (Fig. 4d; two-way ANOVA, hemisphere effect: F_1, 6_ = 7.46, *p* = 0.034, partial *η*^2^ = 0.55, fraction effect: F_6, 36_ = 1.76, *p* = 0.135, partial *η*^2^ = 0.23, interaction: F_6, 36_ = 5.66, *p* < 0.001, partial *η*^2^ = 0.49). These behavioral and biochemical data show that the target neurons expressing IR84a/IR8a based on the viral vector gene expression system respond to peripherally administered ligand precursors in vivo.

### Visualization of radiolabeled ligand binding on IR84a/IR8a-expressing neurons following peripheral administration of the methyl ester precursor

**Fig. 5:**
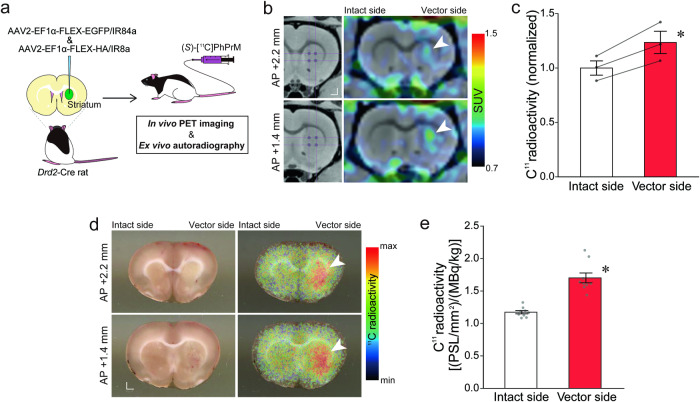
Visualization of the bindings of (*S*)-PhPr on the IR84a/IR8a expressing neurons following peripheral administration of the methyl ester precursor. **a** Schematic representation of brain imaging. The *Drd2*-Cre rats received the unilateral injection of AAV2 vectors that induce IR84a and IR8a in a Cre-dependent manner. These rats were administered (*S*)-[^11^C]PhPrM into the tail vein and used for in vivo PET imaging and ex vivo ARG. **b** Representative coronal PET images with ^11^C overlaid on the magnetic resonance T1-weighted images of the viral vector-treated *Drd2*-Cre rat. The corresponding viral vector injection sites are indicated in the left panel. **c** Plot of ^11^C radioactivity quantified from the PET data and normalized with the mean value of the intact sides, compared between the viral vector-treated and intact sides of the striatum (*n* = 3 rats). *p < 0.05. **d** Representative ARG images with ^11^C for the coronal sections of the vector-treated *Drd2*-Cre rat. The corresponding bright-field images of the sections were indicated in the left panel. The arrowheads indicate the high level ^11^C radioactivity in the striatal regions. **e** Plot of ^11^C radioactivity quantified from the ARG data compared between the viral vector-treated and intact sides of the striatum (*n*s = 9 sections from 3 rats). *p < 0.05. Scale bar: 1 mm (**b, d**). Data are presented as mean with accompanying error bar (SEM) and individual data points superimposed (**c, e**).

Striatal iSPNs expressing IR84a/IR8a using the viral vector system revealed the responsiveness to the peripheral administration with PhAcM and (*S*)-PhPrM. Finally, we sought to visualize the binding of (*S*)-PhPr, converted in the brain from peripherally administered (*S*)-PhPrM, to the IR84a/IR8a complex in striatal neurons. (*S*)-[^11^C]PhPrM was administered intravenously into the lateral tail vein of the *Drd2*-Cre rats that had been microinjected unilaterally with a cocktail of AAV2-EF1α-FLEX-EGFP/IR84a and AAV2-EF1α-FLEX-HA/IR8a vectors into the striatum, and to image ^11^C in the striatum in vivo using positron emission tomography (PET) and ex vivo using autoradiography (ARG) (Fig. 5a). PET imaging showed a high level of ^11^C radioactivity in the viral vector-injected side of the striatal regions of the (*S*)-[^11^C]PhPrM-administered rats (Fig. 5b). Based on the data, we quantified and compared ^11^C radioactivity (normalized) between the vector-treated and intact sides of the striatum, and the vector-treated side had greater ^11^C radioactivity than the intact side (Fig. 5c; one-way ANOVA, the main effect of side: F_1, 2_ = 40.65, *p* = 0.024, partial *η*^2^ = 0.95). Similarly, ex vivo ARG revealed a high accumulation of ^11^C radioactivity in the vector-treated side of the striatum of the same animals (Fig. 5d), and the vector-treated side had greater ^11^C radioactivity than the intact side (Fig. 5e; one-way ANOVA, the main effect of side: F_1, 16_ = 44.23, *p* < 0.001, partial *η*^2^ = 0.73). Since metabolite analyses by TLC (Supplementary Fig. 5) showed that most of the (*S*)-[^11^C]PhPrM was converted to (*S*)-[^11^C]PhPr in the brain within 30 min after the administration, the ^11^C radioactivity visualized in the PET and ARG was considered to be derived from (*S*)-[^11^C]PhPr, but not from (*S*)-[^11^C]PhPrM. Thus, these data support the binding of the ligand that was converted from the systemically administered precursor to the IR84a/IR8a complex in striatal neurons.

## Discussion

This study aimed to improve the previously reported mammalian neural activation technology employing insect IRs^21^ in three ways: first, through development of a pro-drug system that is administered peripherally and then transferred to the central nervous system to induce activation in the target cells expressing IR84a and IR8a; second, a viral vector system that induces expression of the IR84a/IR8a complex in target cell populations; and third, to generalize the functional application of IRNA to other neuron types and mammalian species. Since it was difficult to deliver PhAc into the brain across the BBB, intracranial microinjection of the ligand was necessary to activate the IR84a/IR8a expressing cells in the brain^21^. We therefore developed a strategy to activate the target neurons by esterifying PhAc to make it lipophilic, which facilitates the delivery through the BBB by passive diffusion and reverts it to the active form by esterase activities in the brain^41^. It should be noted that the conversion would also occur in blood, in which esterase activities have previously been reported^42^. Moreover, we generated viral vectors that induce IR84a/IR8a expression according to the Cre-loxP system, and microinjected them into the striatum of the *Drd2*-Cre rats. We demonstrated a highly specific IR84a/IR8a expression in striatal iSPNs, an excitatory response of iSPNs to PhAc in the acute brain slice preparation, behavioral and biochemical responses of iSPNs to PhAcM in vivo, and accumulation of labeled ligands visualized in vivo and ex vivo at the vector injection site, demonstrating the utility of the viral vector system in expressing a functional IR84a/IR8a complex to Cre-expressing cells of Tg animals.

In comparison with the effects of PhAcM (Fig. 1b, c), (*S*)-PhPrM revealed delayed noradrenergic activation in the Tg mice, both in the electrophysiological activity of the LC neurons (Fig. 2b, c) and the NE release in the terminal region (Fig. 2d). A similar delayed response of the IR84a/IR8a-expressing neurons to (*S*)-PhPrM was observed in the striatal neurons of the rats, in which the GABA release in the terminal region of the IR84a/IR8a-expressing iSPNs that was rapidly elevated following PhAcM administration rose slowly following (*S*)-PhPrM (Fig. 4c, d). However, (*S*)-PhPr induced a rapid, excitatory responses in IR84a/IR8a-expressing neurons in the acute LC slice of the Tg mice (Supplementary Fig. 3a–c). The delayed effect of (*S*)-PhPrM administration may be attributable to the complex metabolic processes, such as the precursor translocation into the brain and active form conversion. In particular, conformational interference in the active site of esterase is known to reduce the efficiency of the hydrolysis^43^, and the changed hydrolysis may affect the time course of (*S*)-PhPr bioavailability in the brain regions. In addition, the increase in firing rate of the IR84a/IR8a expressing LC-NE neurons following peripheral administration of PhAcM observed in the present study (Fig. 1b) was milder than the effect on the firing rate of the same neuronal population by the intracranial microinjection of PhAc in our previous reports^21^. The milder effect of PhAcM administration may be explained by lower concentrations of PhAc in the brain region provided from the ligand precursor. It remains to be clarified the extent of conversion from the ligand precursor to the active ligand in the target brain regions.

In our previous report, we used the IRNA technology to clarify the role of LC-NE neurons in emotional memory processing^21^. The present study extends the utility of IRNA as a tool for elucidating the neural basis of behavior by analyzing the role of striatal iSPNs in movement control. When specific activation of iSPNs was induced by the IRNA technology in the unilateral striatum, contraversive rotations were apparent after systemic administration of cocaine. A report that systemic amphetamine administration after selective ablation of iSPNs in the unilateral striatum produced ipsilateral rotation of mice^44^ is in line with the results of the present study. With respect to spontaneous movement, unilateral activations of striatal iSPNs by optogenetic^45–47^ or DREADD-based chemogenetic techniques^48^ were reported to induce ipsiversive rotations in mice. Therefore, the function of iSPNs in the movement control seems to change between spontaneous and drug-induced conditions. Moreover, several studies investigating the effects of unilateral striatal excitotoxic lesions and subsequent psychostimulant administration in rats reported no rotation or rotations in both directions^49–51^. The reliability and direction of rotations were dependent on the topography of the lesion sites within the striatum^52,53^. In the present study, activation of iSPNs was induced in the relatively caudolateral region of the striatum (Supplementary Fig. 8a). Systematic studies on the effects of remote manipulation of iSPN activities in various striatal subregions on drug-induced and spontaneous behaviors are needed in the future.

The IRNA technology is based on insect-derived environmental sensing receptors^54,55^, a highly divergent subfamily of iGluR, which has not been developed as chemogenetic tools. The invertebrate origin of IRNA’s genetic components allows us to omit the process of producing knockout background animals^3,56^, as is the case of the chemogenetic systems based on P2X^19^ and TRP channels^57,58^. Furthermore, compared to G protein-coupled receptor-based chemogenetic systems that depend on the intrinsic second messenger pathways within the targeted cells, the operating mechanism of IRNA is far less complex. Accurate benchmark comparisons between the existing chemogenetic systems and the IRNA technology are difficult since each of the approaches so far has been used in its own distinct experimental paradigm, and systematic comparison of the effects in a similar paradigm targeting the same neuronal population is a future challenge. Nonetheless, the IRNA technology is expected to open the way for remote manipulation of targeting cells that have been difficult to approach with existing technologies^21,59^.

Since molecules that can be sensed vary dramatically by changing the subunit composition of the IR complex^24,25,60,61^, the use of receptor complexes other than the IR84a/IR8a complex is feasible for the application of IRNA technology. In addition, searching for actuator molecules for IR84a/IR8a with higher affinity than PhAc, PhAl, and (*S*)-PhPr would also lead to a more efficient chemogenetic system. Nevertheless, having shown that IRNA works in several cell types in two different mammalian systems, the result s of our study indicate the potential of this system in future as a chemogenetic-based therapeutic strategy for neurological and neuropsychiatric diseases^62,63^.

## Materials and methods

### Animals

We have complied with all relevant ethical regulations for animal use. Tg mice including B6.Cg-Tg(TH-GFP-IR84a/IR8a)2-1Koba^21^ and B6.B6D2-Tg(Th-EGFP)21-31Koba^64^ (Riken BioResource Research Center) were bred with wild-type C57BL/6 J mice (CLEA Japan) to produce the heterozygous and wild-type offspring. Regarding the rats, LE-Tg(Drd2-cre)490-9Koba Tg rats^40^ (The National BioResource Project for the Rat in Japan) were mated with wild-type Long Evans rats (Charles River Laboratories), to obtain heterozygous and wild-type offspring. Animal care and handling procedures followed the guidelines established by the Laboratory Animal Research Center of Fukushima Medical University and RIKEN Biosystems Dynamics Research. All procedures were approved by the Fukushima Medical University Institutional Animal Care and Use Committee and RIKEN Biosystems Dynamics Research. The mice and rats were maintained on a 12 h light/dark cycle (lights on at 07:00 h) at an ambient temperature of 22 °C. All experimental procedures were conducted during the light period. Mice aged 12–14 weeks old and rats aged 8–16 weeks old were used for the following experiments. Male rats were used in the behavioral and biochemical experiments. In all other experiments, both males and females were used. This mice were housed in groups of three to five, whereas the rats were housed in groups of two to three. They were single-housed after the stereotaxic surgeries for the microdialysis experiments. The assignment of animals to the experimental conditions was random.

### Chemical compounds

Phenylacetic acid (PhAc, 166-01232; Wako Pure Chemical) was dissolved in PBS and used as the ligand of the IR84a/IR8a complex to induce neuronal activation. For peripheral administration, phenylacetic acid methyl ester (PhAcM, 108057; Sigma-Aldrich) and (*S*)-2-phenylpropionic acid methyl ester ((*S*)-PhPrM; Sumika Technoservice) were dissolved in PBS containing 1.5% Tween80 and 2.5% ethanol (vehicle) and administered in the lateral tail vein of the mice and rats (at 5 ml/kg) or intraperitoneally injected in rats (at 5 ml/kg). For intracranial microinjection for mice, (*S*)-2-phenylpropionic acid ((*S*)-PhPr, 279900; Sigma-Aldrich), (*R*)-2-phenylpropionic acid ((*R*)-PhPr, 279897; Sigma-Aldrich) were dissolved in the PBS at a concentration of 0.6% (w/v). For the synthesis of (*S*)-2-phenyl[3-^11^C]propionic acid methyl ester ((*S*)-[^11^C]PhPrM), our previous radiolabeling method was used (Takashima-Hirano, et al., ^32^). (*R*),(*S*)-[^11^C]PhPrM was synthesized from the precursor phenyl-propionate by the reaction of [^11^C]CH_3_I with 1 M tetrabutylammonium fluoride in the presence of anhydrous dimethyl sulfoxide, and subsequent separation of racemic form to (*S*)-[^11^C]PhPrM by a chiral column (CHIRALPAK OJ-RH, DAICEL). The radioactivity at the time of the radiosynthesis was 1,990–3,160 MBq (molar radioactivity 28–73 GBq/μmol), and the chemical and radiochemical purities were over 99%. In the behavioral experiments, cocaine hydrochloride (Takeda Pharmaceutical) dissolved in saline was intraperitoneally administered at 15 mg/kg in rats.

### TLC analysis

Each Tg mouse’s brain was removed under deep anesthesia combined with pentobarbital and isoflurane 30 min after intravenous administration of the (*S*)-[^11^C]PhPrM (42 MBq/0.1 ml). The brain tissue was homogenized with a five-fold volume of physiological saline on ice and then vortexed with an equal volume of acetonitrile. The samples were centrifuged (9700 × *g*, for 2 min, at 4 °C), and 2 μl of supernatant obtained was spotted on the origin point of a reversed-phase plate (RP-18, Merck Millipore) and developed with a mixture solution of acetonitrile, distilled water, and formic acid (65:34:1). To further determine whether the metabolite detected by radio-TLC was (*S*)-PhPr, which is the active form to bind IR receptors, we applied with unlabeled (*S*)-PhPrM and (*S*)-PhPr solutions to the origin point on a reversed-phase plate coated with fluorescent indicator (RP-18 F254S, Merck Millipore). The developed spots were detected with ultraviolet light (254 nm).

### Viral vector preparation

Lentiviral vectors pseudotyped with vesicular stomatitis virus glycoprotein (VSV-G)^21,34^ were prepared. V5-tagged IR84a, hemagglutinin (HA)-tagged IR8a, and GFP were connected by 2 A peptide sequence (termed EGFP-2A-V5/IR84a-2A-HA/IR8a), and the GFP-tagged IR84a and IR8a genes were connected by 2 A peptide sequence to construct the FLEX system (FLEX-EGFP/IR84a-2A-IR8a). The transfer plasmids contained the EGFP-2A-V5/IR84a-2A-HA/IR8a or FLEX-EGFP/IR84a-2A-IR8a downstream of the mouse stem cell virus promoter. The envelope plasmid carried VSV-G cDNA under the control of the cytomegalovirus enhancer/chicken β-actin promoter. HEK293T cells (ATCC CRL-11268) were transfected with transfer, envelope, and packaging plasmids using calcium phosphate precipitation. Viral vector particles were pelleted by centrifugation at 6000 × *g* for 16–18 h and suspended in phosphate-buffered saline (PBS); different concentrations (55% and 20%) of sucrose in PBS and particle solution were successively layered from the bottom of the ultracentrifuge tube. The tubes were centrifuged at 100,000 × *g* for 2 h using a SW 55Ti swinging-bucket rotor (Beckman Coulter).

Adeno-associated virus (AAV) vector^33,35^ was prepared using the AAV Helper-Free system (Agilent Technologies). HEK293T cells were transfected with plasmids encoding transfer genes (FLEX-IR84a/EGFP and FLEX-HA/IR8a) downstream of the elongation factor 1α promoter, adeno-helper genes, and adenovirus genes required for AAV replication and encapsulation by a calcium phosphate precipitation method. After 48 h of transfection, the cells were collected and lysed, and crude viral vector lysate was purified with the first round of CsCl gradient ultracentrifugation by using a NVT 65 near-vertical tube rotor (Beckman Coulter) with a quick-seal tube at 287,000 × *g* for 24 h at 16 °C. The needle was inserted into the bottom of the tube to collect drop solution of 1.0 mL (1–10 fractions). To confirm the peak fractions, real-time quantitative PCR was performed using each fraction as a template. The second round of CsCl gradient ultracentrifugation was performed using an SW 55Ti rotor (Beckman Coulter) with a thinwall polypropylene tube at 287,000 × *g* for 24 h at 16 °C. A drop solution of 0.5 mL was collected from the tube, and the peak fractions were confirmed by the real-time quantitative PCR, as described above. The fractions containing vector particles were collected and applied to three rounds of dialysis against PBS using a Slide-A-Lyzer G2 dialysis cassette (MWCO 10,000; Thermo Fisher Scientific). The dialyzed solution was finally concentrated by centrifugation through a Vivaspin Turbo 4 membrane filter (MWCO 10,000; Sartorius) at 3500 × *g* for 30–45 min at 4 °C.

Viral genome titer was determined by quantitative PCR using a TaqMan system (Thermo Fisher Scientific). PCR amplification was performed using a StepOne real-time PCR system (Applied Biosystems) as follows: one cycle of 3 min and 15 s at 95 °C; and 40 cycles for 30 s at 60 °C for duplicate samples. Standard curves were prepared based on serial dilutions of the viral DNA control template from 4.0 × 10^4^ to 4.0 × 10^7^ genome copies/ml. NG108-15 cells (ATCC HB12317, ~4 × 10^4^ cells in a 3.5-cm dish) were transduced with the lentiviral vector encoding EGFP-2A-V5/IR84a-2A-HA/IR8a with a functional titer of 2.3 × 10^12^ genome copies/ml (10 µl), and used for electrophysiological experiments.

### Viral vector injection

*Drd2*-Cre rats were anesthetized with isoflurane (4% induction and 1.5% maintenance) and secured in a stereotaxic frame (SR-6R-HT, Narishige). Lenti-FLEX-EGFP/IR84a-2A-IR8a vector (4.36 × 10^12^ genome copies/ml for histological, behavioral, and biochemical experiments; 1.68 × 10^12^ genome copies/ml for ex vivo electrophysiological experiment), Lenti-FLEX-EGFP vector (4.97 × 10^13^ genome copies/ml for ex vivo electrophysiological experiment), or a 1:1 mixture of AAV2-EF1α-FLEX-EGFP/IR84a and AAV2-EF1α-FLEX-HA/IR8a (2.16 × 10^12^ genome copies/ml and 3.31 × 10^12^ genome copies/ml, respectively, for in vivo PET and ex vivo ARG experiments) were injected into the dorsal striatum through a glass microinjection capillary connected to a 10-μl gas-tight syringe (1801N, Hamilton) set in a microinfusion pump (ESP-32; Eicom). Injection was performed at a constant rate of 0.1 μl/min for 5 min according to the following anteroposterior (AP), mediolateral (ML), and dorsoventral (DV) coordinates from the bregma and dura (mm) of the rat brain^65^: +0.15/ ± 3.8/ − 4.4 (Site 1), +0.15/ ± 3.8/ − 3.4 (Site 2), +0.15/ ± 4.0/ − 4.4 (Site 3), +0.15/ ± 4.0/ − 3.4 (Site 4) (bilateral) for the histological experiments (Fig. 3a); and +0.15/ ± 3.8/ − 4.4 (Site 1), +0.15/ ± 3.8/ − 3.4 (Site 2), +0.15/ ± 4.0/ − 4.4 (Site 3), +0.15/ ± 4.0/ − 3.4 (Site 4), +0.15/ ± 4.2/ − 4.4 (Site 5), +0.15/ ± 4.2/ − 3.4 (Site 6), −0.35/ ± 3.7/ − 4.7 (Site 7), −0.35/ ± 3.7/ − 3.7 (Site 8), -0.35/ ± 3.9 − 4.7 (Site 9), −0.35/ ± 3.9/ − 3.7 (Site 10), −0.35/ ± 4.1/ − 4.7 (Site 11), −0.35/ ± 4.1/ − 3.7 (Site 12) for the ex vivo electrophysiological experiment (bilateral, Fig. 3b–d) and biochemical experiments (unilateral, random assignment of the viral vector injection to either the left or right hemisphere, Fig. 4b–d); +1.44/2.0/ − 6.0 (Site 1), +1.44/2.0/ − 5.0 (Site 2), +1.44/3.0/ − 6.0 (Site 3), +1.44/3.0/ − 5.0 (Site 4), +0.6/2.5/ − 6.0 (Site 5), +0.6/2.5/ − 5.0 (Site 6), +0.6/3.5/ − 6.0 (Site 7), +0.6/3.5/ − 5.0 (Site 8) (unilateral) for in vivo PET and ex vivo ARG imaging (Fig. 5b, c).

### Histology

Rats were anesthetized with sodium pentobarbital (100 mg/kg, ip.) and perfused transcardially with PBS, then fixed with 4% paraformaldehyde in 0.1 M phosphate buffer (pH 7.4). Sections (50-μm thick) were cut from the fixed brains with a vibrating blade microtome (VT1000S, Leica), and then incubated with 10% normal donkey serum, followed by mixtures of a primary antibodies for Cre (mouse, 1:200; MAB3120, Sigma-Aldrich), A_2A_R (goat, 1:200; A2A-Go-Af700, Frontier Institute), and D1R (guinea pig, 1:200; D1R-GP-Af500, Frontier Institute) (Supplementary Fig. 7), or GFP (chicken, 1:1,000; ab13970, Abcam), IR8a (guinea pig, 1:1,000; Abuin et al. ^25^), and A_2A_R, or GFP, IR8a, and D1R (goat, 1:200, D1R-Go-Af1000, Frontier Institute) (Fig. 3a), or GFP and IR8a (Supplementary Fig. 8a). Alexa Fluor 647-conjugated donkey anti-mouse antibody (A-31571, Thermo Fisher Scientific), Alexa Fluor 488-conjugated donkey anti-goat antibody (A-11055, Thermo Fisher Scientific), Cy3-conjugated donkey anti-guinea pig antibody (706-165-148, Jackson ImmunoResearch Laboratories), Alexa Fluor 488-conjugated donkey anti-chicken antibody (703-545-155, Jackson ImmunoResearch Laboratories), and Alexa Fluor 647-conjugated donkey anti-goat antibody (A-21447, Thermo Fisher Scientific) were used as species-specific secondary antibodies for detecting Cre, A_2A_R, D1R, GFP, and A_2A_R, respectively (all dilutions were 1:200). Fluorescent images were obtained with a confocal laser-scanning microscope (A1, Nikon) or all-in-one fluorescence microscope (BZ-X810, Keyence) equipped with proper filter cube specifications.

For cell counts of triple-fluorescence histochemistries for GFP (IR84a), IR8a, and A_2A_R, and GFP, IR8a, and D1R in rats (Fig. 3a), 4–5 sections through the striatum were prepared from each hemisphere and used for immunostaining. The number of immuno-positive cells in the region of interest was counted by using a computer-assisted imaging program (Photoshop 2021; Adobe). The number of cells of each type from 5–7 visual fields of the same hemisphere (200 × 200 μm each, a total of 50–80 identified cells) was counted, and the means of the four hemispheres are shown in Supplementary Table 1. The IR84a/IR8a double-expression efficiency in the A_2A_R- and D1R-positive neurons was calculated by dividing the number of IR84a/IR8a double-positive neurons by the number of triple-positive neurons. The IR84a/IR8a double-expression specificity was calculated by dividing the number of triple-positive neurons by the number of IR84a/IR8a double-positive neurons.

### Electrophysiology

In vivo extracellular single-unit recording^21^ was performed with the Tg mice (Fig. 1b and Fig. 2b, c). Mice were anesthetized with 1.5% isoflurane and placed in the stereotaxic frame (SR-5M, Narishige) with ear bars and a mouth-and-nose clamp. Anesthesia was maintained with 0.5–1.0% isoflurane based on the electroencephalogram monitoring with body temperature at 37–38 °C using a heating pad. The scalp was opened, and a hole was drilled in the skull above the LC with the coordinates (in mm) AP − 1.2 and ML ± 0.9 from lambda according to the mouse stereotaxic atlas^66^. Two skull screws were placed over the occipital bone, and another skull screw was placed on the frontal bone. One of the two screws over the occipital bone was used as a reference for the recording. The firing activity of LC neurons was recorded using a recording electrode (tip diameter: 2–3 μm, impedance: 15–20 MΩ, Harvard Apparatus), which was lowered into the LC, dorsoventral (DV) − 2.2 to −3.5 mm from the dura, and the recording was performed using Spike2 (Cambridge Electronic Design) at a sampling rate of 20 kHz. NE cells in the LC were identified based on a slow tonic firing (<7 Hz) with long spike duration (>0.8 ms)^21,67^. A solution of PhAcM (20 mg/kg), (*S*)-PhPrM (20 mg/kg) or vehicle was administered via the lateral tail vein at 5 ml/kg. The baseline firing rate (pre) was recorded during a 180-s period immediately before drug administration. Ten mins after the drug administration, the effects were assessed for 10 min (post for PhAcM, Fig. 1b) or 90 min (post for (*S*)-PhPrM, Fig. 2b, c).

Ex vivo electrophysiological experiments^21^ were performed with the coronal brain slices containing the LC (in mice, Supplementary Fig. 3a–f) or striatum (in rats, Fig. 3b–d). Animals were anesthetized with 1.5% isoflurane and the brain slices containing target area were cut (300-μm thick) using a microslicer (PRO7, Dosaka) in ice-cold oxygenated cutting Krebs solution of the following composition: choline chloride, 120 mM; KCl, 2.5 mM; NaHCO_3_, 26 mM; NaH_2_PO_4_, 1.25 mM; D-glucose, 15 mM; ascorbic acid, 1.3 mM; CaCl_2_, 0.5 mM; and MgCl_2_, 7 mM. The slices were then transferred to a holding chamber containing a standard Krebs solution of the following composition: NaCl, 124 mM; KCl, 3 mM; NaHCO_3_, 26 mM; NaH_2_PO_4_, 1 mM; CaCl_2_, 2.4 mM; MgCl_2_, 1.2 mM; and D-glucose, 10 mM (pH 7.4) when bubbled with 95% O_2_-5% CO_2_. The slices were incubated in the holding chamber at room temperature (21–26 °C) for at least 1 h before recording. Neurons were visualized with a 60× water immersion objective attached to an upright microscope (BX50WI, Olympus Optics), and fluorescence was visualized using the appropriate fluorescence filter. Patch pipettes were made from standard-walled borosilicate glass capillaries (Harvard Apparatus). For the recording of membrane potentials, a K-gluconate-based internal solution of the following composition was used: K-gluconate, 120 mM; NaCl, 6 mM; CaCl_2_, 5 mM; MgCl_2_, 2 mM; K-EGTA, 0.2 mM; K-HEPES, 10 mM; Mg-ATP, 2 mM; and Na-GTP, 0.3 mM (pH adjusted to 7.4 with 1 M KOH). Whole-cell recordings were made from LC neurons with fluorescence using a patch-clamp amplifier (Axopatch 200B, Molecular Devices). The effects of drugs (*S*)-PhPr and (*R*)-PhPr for the mouse LC neurons (Supplementary Fig. 3a–f), as well as that for PhAc for the rat striatal neurons (Fig. 3b–d) on membrane potential were assessed after they had reached a steady state (starting point), and the mean firing frequency and membrane potential were calculated during a 30-s test period before the drug application (pre) and after the starting point (post).

In vitro electrophysiological experiments^68–70^ were performed with IR84a/IR8a-expressing NG108-15 cells (Supplementary Fig. 4). All electrophysiological measurements were performed at room temperature (22–25 °C) using an Axopatch 200B patch-clamp amplifier (Molecular Devices) and pCLAMP 10 software (Molecular Devices). The pipette solution contained KCl 107 mM, MgCl_2_ 2 mM, CaCl_2_ 1 mM, EGTA 10 mM, HEPES 10 mM, and ATP 0.3 mM (pH 7.2 with KOH), and the extracellular solution contained NaCl 138 mM, KCl 5.6 mM, MgCl_2_ 1 mM, HEPES 10 mM, and CaCl_2_ 2.6 mM (pH 7.4 with NaOH). The effects of 1.0, 5.0, and 10.0 mM (*S*)-PhPr on the IR84a/IR8a complex were evaluated using the standard whole-cell technique, measuring the current with a holding potential of − 70 mV. Data were analyzed using Clampfit software (Molecular Devices).

After the electrophysiological experiments, the recording sites were marked by iontophoretic injection of 2% pontamine sky blue. The mice were deeply anesthetized with sodium pentobarbital and perfused transcardially with PBS, followed by 10% formalin. Brain sections were stained with neutral red for the verification of recording sites. The post-mortem histological analysis verified deposits of recording sites in the LC (Supplementary Fig. 2a, Supplementary Fig. 6a).

### Microdialysis

To monitor changes in the NE_[ext]_ in the ACC^21^, mice were anesthetized with 1.5% isoflurane and underwent stereotaxic surgery for a dialysis probe aimed at unilateral ACC with 25-gauge guide cannula (AG-X series, Eicom). A group of mice (for Supplementary Fig. 3g, h) also underwent stereotaxic surgery for drug microinjection into the ipsilateral LC with a 30-gauge guide cannula (AG-X(T) series, Eicom). The coordinates (mm) from the bregma and dura were AP + 0.7, ML ± 0.3, and DV − 0.4 for the ACC dialysis probe and AP − 0.75, ML ± 0.65, and DV − 2.5 for the LC microinjection, according to the mouse stereotaxic atlas^66^. Two or three days later, the stylet in the cannula was replaced with an active membrane dialysis probe (1.0 mm in length, 0.22 mm in outer diameter, FX-I series, Eicom) that was connected to a 2,500 μl syringe filled with artificial cerebrospinal fluid (aCSF) with the following composition: NaCl, 148 mM; KCl, 4.0 mM; MgCl_2_, 0.85 mM; and CaCl_2_, 1.2 mM. For the intra-LC injection, the stylet in the LC cannula was replaced with a 35-gauge internal cannula (1 mm beyond the tip of the implanted guide cannula, AMI-X(T) series, Eicom) connected to a 10-μl Hamilton syringe. aCSF was pumped through the probe at a rate of 1.0 μl/min for 2 h, and then dialysate samples were collected in sampling vials every 30 min using a refrigerated fraction collector (EFC-82, Eicom). Each sampling vial was pre-loaded with 10 μl of 20 mM phosphate buffer, including 25 mM EDTA-2Na and 0.5 mM ascorbic acid (pH 3.5) as antioxidants. Three baseline samples were collected to measure a tonic level of NE. This was followed by tail vein injection of PhAc (Supplementary Fig. 1a), PhAcM (Fig. 1c), or (*S*)-PhPrM (Fig. 2d), or intracranial LC microinjection (at a flow rate of 0.05 μl/min for 5 min) of 0.6% (*S*)-PhPr or 0.6% (*R*)-PhPr (Supplementary Fig. 3g). Four samples were collected thereafter to assess the time course for changes in NE level after these interventions. The amount of NE in each fraction was determined by a high-performance liquid chromatography (HPLC) system (CA-50DS, 2.1 mm × 150 mm, Eicom, with the mobile phase containing 5% methanol in 100 mM sodium phosphate buffer, pH 6.0), equipped with an electrochemical detector (ECD-300, Eicom). Results are expressed as percentages of the baseline concentrations (analyte concentration × 100/mean of the three baseline samples).

To monitor changes in the GABA_[ext]_ in the GPe, the viral vector-treated *Drd2*-Cre rats that had been subjected to behavioral experiments were anesthetized with 1.5% isoflurane and underwent stereotaxic surgery for a dialysis probe aimed at bilateral GPe with 25-gauge guide cannula. According to the rat stereotaxic atlas, the coordinates (mm) from the bregma and dura were AP − 0.9, ML ± 3.0, and DV − 5.1^65^. Seven to 10 days later, the stylet in the cannula was replaced with an active membrane dialysis probe, and aCSF was perfused through the probe at a rate of 1.0 μl/min for 2 h, and then dialysate samples were collected in sampling vials every 30 min using a refrigerated fraction collector. Each dialysate sample was reacted with 10 μl of 20 mM o-phthalaldehyde, including 0.2% 2-mercaptoethanol, for 2.5 min and then injected into HPLC at a volume of 30 μl (SC-50DS, 2.1 mm × 150 mm, Eicom, with the mobile phase containing 50% methanol in 50 mM sodium phosphate buffer, pH 6.0) equipped with an electrochemical detector. Three baseline samples were collected to measure GABA tone. This was followed by a lateral tail vein injection of 10 mg/kg PhAcM (Fig. 4c) or 20 mg/kg (*S*)-PhPrM (Fig. 4d). Four samples were collected thereafter to assess the time course for change in GABA levels after these interventions. Results are expressed as percentages of the baseline concentrations (analyte concentration × 100/mean of the three baseline samples).

After the microdialysis experiments, the animals were deeply anesthetized with sodium pentobarbital and perfused transcardially with PBS, followed by 10% formalin. Brain sections were stained with cresyl violet for the verification of placement sites. The post-mortem histological analysis verified deposits of dialysis probe sites in the ACC of the mice (Supplementary Fig. 1c, Supplementary Fig. 2b, Supplementary Fig. 3h, and Supplementary Fig. 6b) and the GPe of the rats (Supplementary Fig. 8b) as well as microinjection sites in the LC of mice (Supplementary Fig. 3h).

### Behavioral analysis

The rotational behavior of the rats was examined in a transparent, acrylic plastic cylinder (30 cm in diameter, 55 cm in height) with a hemispheric bottom as the apparatus. A three-day test was conducted after 14 days of recovery from the stereotaxic surgery for viral vector administration, during which animals were handled and acclimated to the apparatus. On day 1 of the two-day test, the rats were given 10 mg/kg PhAcM and 15 mg/kg cocaine i.p. consecutively at 10-min intervals and then collared and placed in the apparatus for 60 min to monitor their rotations. The collar was connected, via a stainless wire, to a rotary encoder (E6A2-CWZ3C, Omron), which determines clockwise and anti-clockwise rotations of animals. The rotation data was transferred to a personal computer via an interface (USB-6501, National Instruments) and recorded using a Matlab simulink-based application (Matlab R2019b, Mathworks). No explicit treatments were carried out on day 2. The procedure for day 3 of the test was performed 48 h after day 1 similarly, except that a vehicle was administered instead of PhAcM. Rotational behavior recorded during the 60-min test sessions was analyzed for ipsiversive and contraversive ones of the viral vector-treated hemisphere every 5-min bins. The range of expression of IR84a (GFP)/IR8a double-positive cells in the rat striatum was visualized in the post-mortem histological analysis (Supplementary Fig. 8a).

### PET imaging and ex vivo ARG

Rats were placed under isoflurane anesthesia with a plastic catheter (26 G) in their tail vein for PET tracer administration. The rats were then set in an animal PET scanner (microPET Focus-220, Siemens Medical Solutions) for a head scan. A bolus injection of (*S*)-[^11^C]PhPrM was performed at 68.7–75.2 MBq/0.3 ml in physiological saline for 10 s. After dosing, saline was flushed with 0.5 ml of saline. The emission scan was done at the same time as the dose for 60 min. During the PET scanning, the animal was kept under isoflurane anesthesia (2.2–3.0%). The acquired emission data reconstructed the PET images using a filtered back-projection algorithm with no scattered and attenuation corrections. The regional ^11^C radioactivity on the PET images was analyzed using an imaging software (PMOD ver. 3.9, PMOD Technologies LLC). The volumes of interest in the vector-injected and intact opposite side were drawn on the PET images superimposed with the MR-T2 images as an anatomical reference. The ^11^C radioactive signals on the PET images were represented by the standardized uptake value (SUV) as calculated the regional binding intensity (Bq/ml) by dividing the injected dose (Bq/g).

For ex vivo ARG, the brain tissue samples of the rats used for PET imaging were removed immediately after euthanasia under deep anesthesia. The brains were sectioned coronally (1–mm thick) using a brain slicer under ice-cold conditions, and the resulting sections were placed in a chamber with plastic film attached and exposed to an imaging plate (MS-2040, Fuji Film) for 40 min. The exposed imaging plate was taken with an image analyzer (FLA-7000IR, Fuji Film). The photographs of the brain sections were also acquired with a digital scanner as an anatomical reference for brain regions. To quantify ^11^C radioactivity, Multi Gage software (Fuji Film), the regions of interests in the vector-injected and opposite control sides were drawn on ARG images. The intensity of ^11^C radioactivity was expressed as photo-stimulated luminescence (PSL) per area (mm^2^) normalized by dividing the injected dose (MBq/kg).

### Statistics and Reproducibility

Although no statistical methods were used to predetermine the sample size for each measure, we employed similar sample sizes to those reported in previous publications from our labs, which have been generally accepted in the field. In each test, we conducted an experiment in two to five cohorts, each designed to include all experimental groups. All statistical analyses were two-tailed and conducted using SPSS ver. 25 (IBM). The reliability of the results was assessed against a type I error (*α*) of 0.05. For significant main effects identified in one- and two-way analyses of variance (ANOVAs). For significant interactions revealed in two-way ANOVAs, t-tests with Bonferroni correction were used post hoc. Besides, all effect sizes were reported as partial *η*^2^ for main effects and interaction in ANOVAs and as *r* for post hoc paired comparisons. In principle, the main text describes the main effects and interaction of ANOVA,s and subsequent tests are reported in the corresponding figure legends. The star and dagger (*, †) in the figures represent *p*-values of < 0.05.

### Supplementary information

Peer Review File

Supplementary Information

Reporting-summary

## Data Availability

The datasets generated and analyzed in this study are deposited on Mendeley data. 10.17632/b47j739jb7.2https://data.mendeley.com/datasets/b47j739jb7/2 Any remaining information can be obtained from the corresponding author upon reasonable request.
