# Supplementary material for: Chemogenetic activation of mammalian brain neurons expressing insect Ionotropic Receptors by systemic ligand precursor administration
Source: Commun Biol. 2024 May 7;7:547. doi: 10.1038/s42003-024-06223-4 (PMC11076466; doi:10.1038/s42003-024-06223-4)
Supplement: Supplementary file 2 — Supplementary Information [file 42003_2024_6223_MOESM2_ESM.pdf]

Supplementary Information

Chemogenetic activation of mammalian brain neurons expressing insect Ionotropic Receptors by systemic ligand precursor administration

Yoshio Iguchi[1], Ryoji Fukabori[1], Shigeki Kato[1], Kazumi Takahashi[2], Satoshi Eifuku[2], Yuko Maejima[3], Kenju Shimomura[3], Hiroshi Mizuma[4,5], Aya Mawatari[6], Hisashi Doi[6,7], Yilong Cui[8], Hirotaka Onoe[9], Keigo Hikishima[10], Makoto Osanai[11], Takuma Nishijo[12,13], Toshihiko Momiyama[12], Richard Benton[14], and Kazuto Kobayashi[1*]

[1]Department of Molecular Genetics, Institute of Biomedical Sciences, Fukushima Medical University School of Medicine, Fukushima, Japan

[2]Department of Systems Neuroscience, Fukushima Medical University School of Medicine, Fukushima Japan

[3]Department of Bioregulation and Pharmacological Medicine, Fukushima Medical University School of Medicine, Fukushima Japan

[4]Laboratory for Pathophysiological and Health Science, RIKEN Center for Biosystems Dynamics Research, Kobe, Japan

[5]Department of Functional Brain Imaging, Institute for Quantum Medical Science, National Institutes for Quantum Science and Technology, Chiba, Japan

[6]Laboratory for Labeling Chemistry, RIKEN Center for Biosystems Dynamics Research, Kobe, Japan

[7]Research Institute for Drug Discovery Science, Collaborative Creation Research Center, Organization for Research Promotion, Osaka Metropolitan University

[8]Laboratory for Biofunction Dynamics Imaging, RIKEN Center for Biosystems Dynamics Research, Kobe, Japan

[9]Human Brain Research Center, Kyoto University Graduate School of Medicine, Kyoto, Japan

[10]Medical Devices Research Group, Health and Medical Research Institute, National Institute of Advanced Industrial Science and Technology (AIST), Tsukuba, Japan

[11]Department of Medical Physics and Engineering, Division of Health Sciences, Osaka University Graduate School of Medicine, Suita, Japan

[12]Department of Pharmacology, Jikei University School of Medicine, Tokyo, Japan

[13]Department of Molecular Neurobiology, Institute for Developmental Research, Aichi Developmental Disability Center, Kasugai, Japan

[14]Center for Integrative Genomics, Faculty of Biology and Medicine, University of Lausanne, Lausanne, Switzerland

[*]**For correspondence:** kazuto@fmu.ac.jp (KK)

This Supplementary Information PDF contains the following:

Supplementary Figures

Supplementary Figure 1: Peripheral administration of PhAc does not stimulate the IR84a/IR8a-expressing neurons in the brain.

Supplementary Figure 2: Placement sites of glass electrodes for in *vivo* electrophysiology and dialysis probes for *in vivo* microdialysis analysis in Figure 1.

Supplementary Figure 3: The *S*-isomer of PhPr stimulates IR84a/IR8a-expressing neurons.

Supplementary Figure 4: TLC analysis of the tissue sample dissected from the LC area of the Tg mouse brain after (S)-[11C]PhPrM treatment.

Supplementary Figure 5: Placement sites of glass electrodes for *in viv*o electrophysiology and dialysis probes for *in vivo* microdialysis analysis in Figure 2.

Supplementary Figure 6: Specificity of the Cre recombinase expression in iSPNs of the striatum of the *Drd2*-Cre rats.

Supplementary Figure 7: Expression patterns of transgenes and placement sites dialysis probes for microdialysis analysis.

Supplementary Table

Supplementary Table 1: Count of various types of immuno-positive neurons in the viral vector-treated striatum of the *Drd2*-Cre rats.

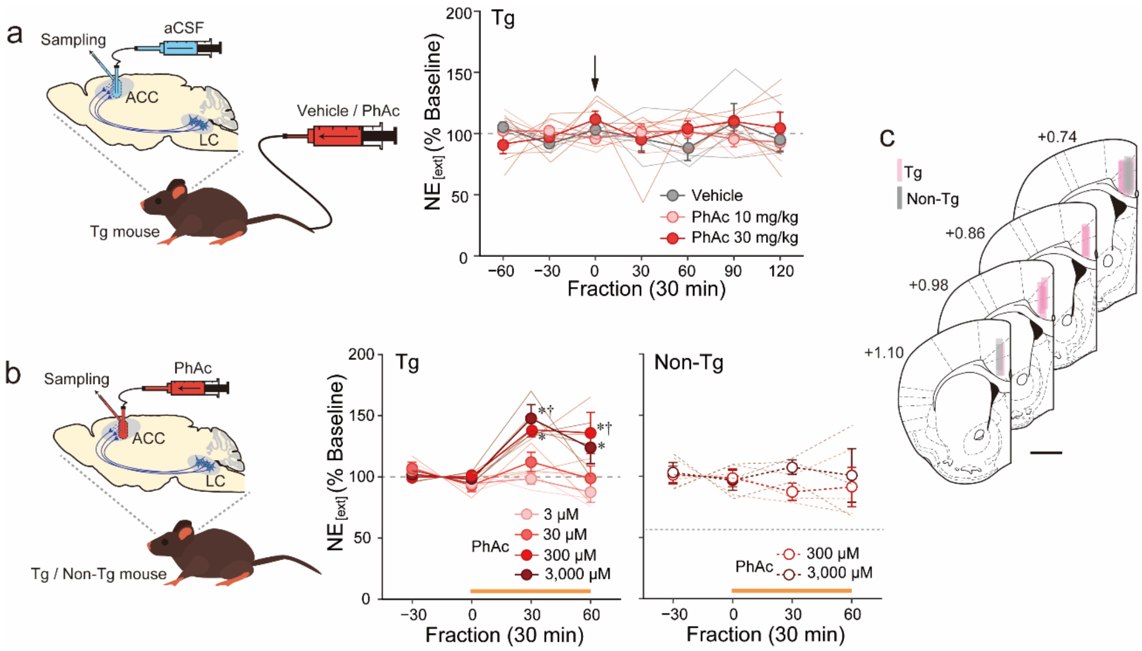

Supplementary Figure 1: Peripheral administration of PhAc does not stimulate the IR84a/IR8a-expressing neurons in the brain.

a Plot of changes in the $NE_{[ext]}$ in the ACC of the TH-IR84a/IR8a (Tg) mice before and after tail vein injection with vehicle or PhAc (10 or 30 mg/kg). $NE_{[ext]}$ is expressed as a percentage of the average baseline levels of each mouse. Arrow indicates the timing of drug injection. $NE_{[ext]}$ did not show any significant changes among drug administrations (*n*s = 4 mice for vehicle and PhAc 10 mg/kg, n = 6 mice for PhAc 30 mg/kg); two-way ANOVA, dose effect: $F_{2, 11} = 0.28$, $p = 0.762$, partial $\eta^2 = 0.05$, fraction effect: $F_{6, 66} = 0.49$, $p = 0.813$, partial $\eta^2 = 0.04$, interaction: $F_{12, 66} = 0.78$, $p = 0.671$, partial $\eta^2 = 0.12$. Data are presented as mean with accompanying error bar (SEM) and individual data points superimposed (**a, b**). **b** Plot of changes in $NE_{[ext]}$ in the ACC of the Tg and non-Tg mice before and after reverse dialysis with PhAc. Horizontal bars indicate the period of PhAc perfusion. A perfusion of different concentrations of PhAc (3 - 3,000 μM) into the dialysis probe elevated $NE_{[ext]}$ in a dose-dependent manner in the Tg mice (n = 3 mice for each condition); two-way ANOVA, concentration effect: $F_{3, 8} = 27.14$, $p < 0.001$, partial η^2 = 0.91, fraction effect: $F_{3, 24} = 7.38$, $p = 0.001$, partial $\eta^2 = 0.48$, interaction: $F_{9, 24} = 2.38$, p = 0.044, partial $\eta^2 = 0.47$; $NE_{[ext]}$ following the 3,000-μM PhAc perfusion was significantly higher than those following the *3- and †30-μM PhAc at the 30-min fraction, $ts_{32} = 4.41$, 3.18, $p < 0.001$, $p = 0.020$, *r*s = 0.62, 0.49, respectively, and that following the *3-μM PhAc at the 60-min fraction, $t_{32} = 3.24$, $p = 0.017$, $r = 0.50$, whereas $NE_{[ext]}$ following the 300-μM PhAc perfusion was significantly higher than that following the *3 μM PhAc at the 30-min fraction, $t_{32} = 3.59$, $p = 0.007$, $r = 0.54$, and those following the *3- and †30-

μM PhAc at the 60-min fraction, ts_{32} = 4.33, 3.35, p < 0.001, p = 0.012, rs = 0.61, 0.51, respectively. $NE_{[ext]}$ did not show any significant changes for the higher concentrations of PhAc (300–3,000 μM) in non-Tg animals (n = 3 for each condition); two-way ANOVA, dose effect: $F_{1, 4} = 0.69$, $p = 0.452$, partial $\eta^2 = 0.15$, fraction effect: $F_{3, 12} = 0.11$, $p = 0.952$, partial $\eta^2 = 0.03$, interaction: $F_{3, 12} = 0.38$, $p = 0.767$, partial $\eta^2 = 0.09$. **c** Site mapping of the dialysis probes in the ACC of the Tg and non-Tg mice. The anteroposterior coordinates (mm) are shown. Scale bar: 1 mm.

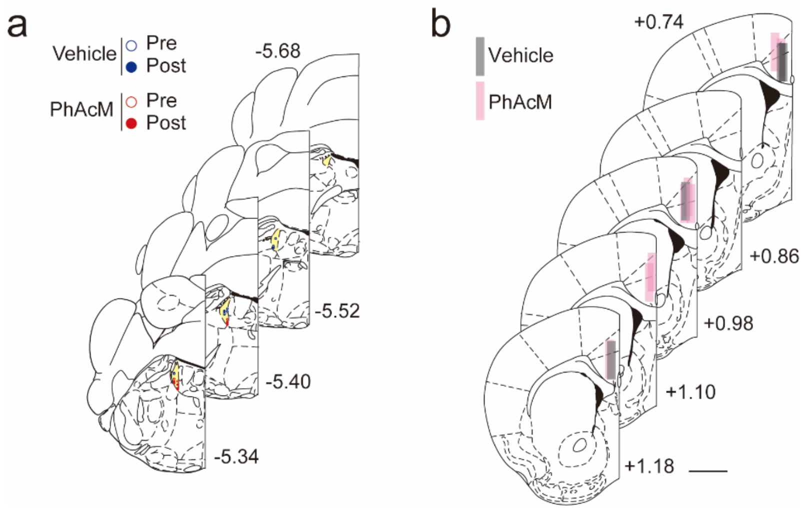

Supplementary Figure 2: Placement sites of glass electrodes for *in vivo* electrophysiology and dialysis probes for *in vivo* microdialysis analysis in Figure 1.
a Site mapping of the tips of glass electrode in the LC of the Tg mice (for Fig. 1b). **b** Site mapping of the dialysis probes in the ACC of the Tg and non-Tg mice (for Fig. 1c). The anteroposterior coordinates (mm) are shown. Scale bar: 1 mm.

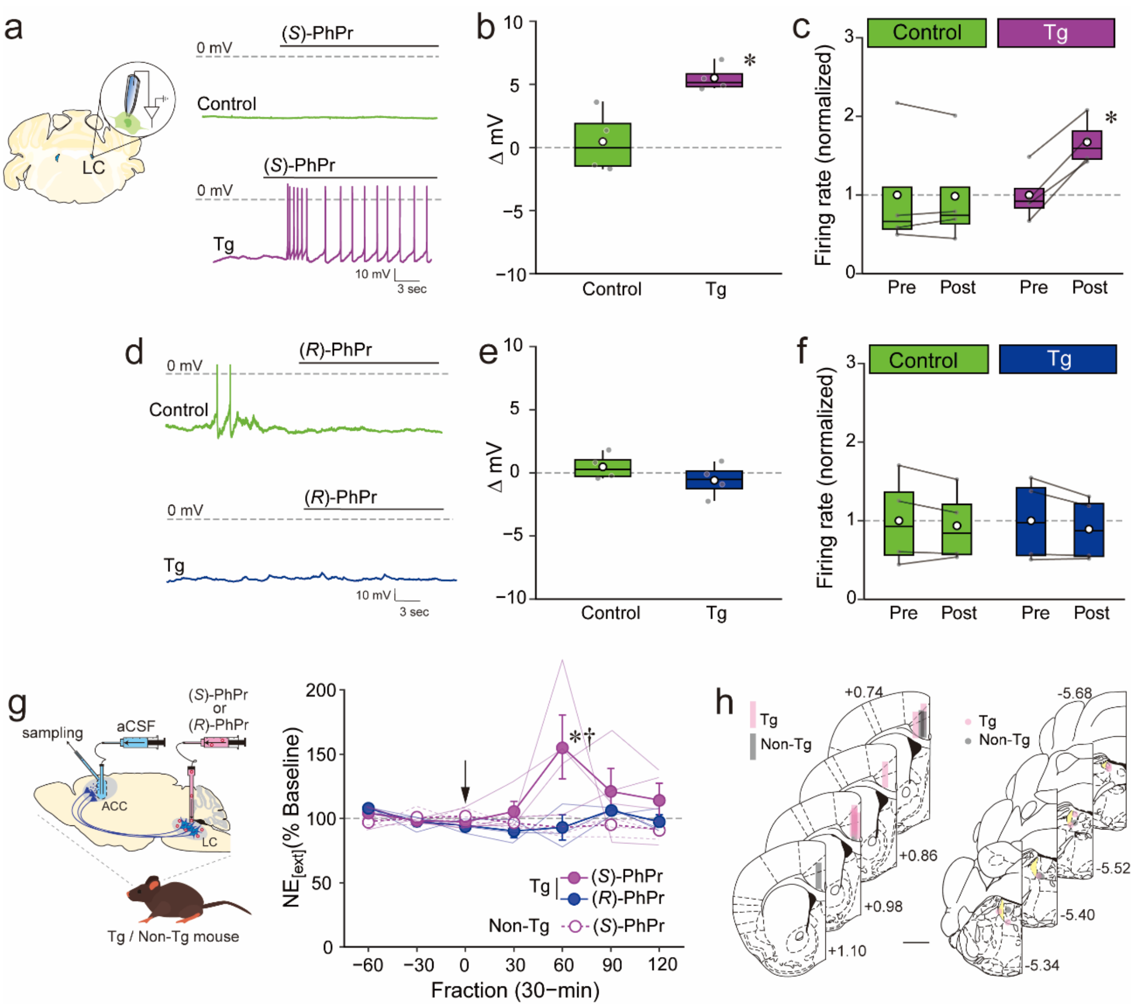

Supplementary Figure 3: The *S*-isomer of PhPr stimulates IR84a/IR8a-expressing neurons.

a Representative whole-cell recordings of the LC neurons of the control (TH-GFP) and Tg (TH-IR84a/IR8a) mice in response to (*S*)-PhPr. **b, c** Plot of changes in the membrane potential (**b**) and firing rate (**c**) with addition of (*S*)-PhPr (0.1% w/v). Data are presented as boxplots with the mean values represented by white circles and individual data points superimposed (**b, c, e, f**). The amount of difference in the membrane potential between pre- and post-(*S*)-PhPr (0.1%) bath application (Δ mV) of the LC-NE neurons in the Tg mice was significantly greater than that of the LC-NE neurons in the control mice (n = 4 neurons for each group); one-way ANOVA, genotype effect: $F_{1,6}$ = 13.65, p = 0.010, partial η^2 = 0.69. The normalized firing rate was also significantly elevated by the (*S*)-PhPr application in the Tg mice but not in the control mice; one-way ANOVA for the Tg mice, genotype effect: $F_{1,3}$ = 109.13, p = 0.002, partial η^2 = 0.97; for the control mice, $F_{1,3}$ = 0.05, p = 0.836, partial η^2 = 0.02. **d** Representative whole-cell recordings of the LC neurons of the control and Tg mice in response to (*R*)-PhPr. **e, f** Plot of changes in the

membrane potential (**e**) and firing rate (**f**) with addition of (*R*)-PhPr (0.1% w/v). Δ mV by (*R*)-PhPr (0.1%) was almost zero for both the control and Tg mice and no significant difference was found between the two conditions (n = 4 neurons for each group); one-way ANOVA, genotype effect: $F_{1, 6}$ = 1.64, p = 0.247, partial η^2 = 0.21. (*R*)-PhPr had no significant impacts on the normalized firing rate of not only control but also Tg mice; one-way ANOVA for the control mice, genotype effect: $F_{1, 3}$ = 0.94, p = 0.403, partial η^2 = 0.24; for the Tg mice, $F_{1, 3}$ = 2.64, p = 0.203, partial η^2 = 0.47. **g** Plot of changes in the extracellular NE level ($NE_{[ext]}$) in the ACC area of the Tg and non-Tg mice before and after microinjection of (*S*)- and (*R*)-PhPr (0.6% w/v) into the LC. (*S*)-PhPr resulted in a slightly delayed but marked increase in $NE_{[ext]}$ in the Tg mice (ns = 4 mice for the two (*S*)-PhPr groups, n = 3 mice for (*R*)-PhPr group); two-way ANOVA, group effect: $F_{2, 8}$ = 26.42, p < 0.001, partial η^2 = 0.89, fraction effect: F6, 48 = 1.21, p = 0.318, partial η^2 = 0.13, interaction: $F_{12, 48}$ = 2.01, p = 0.044, partial η^2 = 0.33; $NE_{[ext]}$ of (*S*)-PhPr in the Tg mice was significantly higher than that of *(*R*)-PhPr in the Tg mice and †(*S*)-PhPr in the non-Tg mice at the 60-min fractions, ts_{56} = 4.82, 5.19, ps < 0.001, rs = 0.54, 0.57, respectively. Data are presented as mean with accompanying error bar (SEM) and individual data points superimposed. **h** Site mapping of the dialysis probes in the ACC and the injection cannula tips near the LC of the Tg and non-Tg mice (for **g**). The anteroposterior coordinates (mm) are shown. Scale bar: 1 mm.

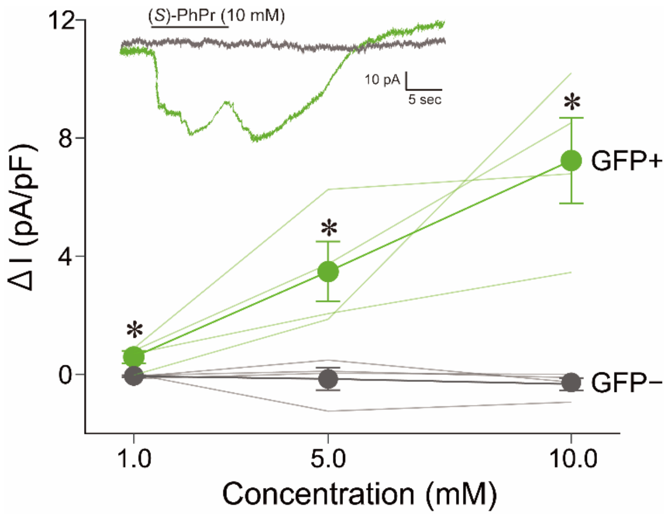

Supplementary Figure 4: Dose responses of IR84a/IR8a-expressing NG108-15 cells to *S*-isomer of PhPr.

Changes in peak currents of IR84a/IR8a-positive (GFP+) and negative (GFP-) cells were divided by each membrane capacitance (13.92 ± 1.92 pF) and reported as changes in peak current densities (ΔI [pA/pF]). ΔI of GFP+ and GFP- cells with addition of (*S*)-PhPr were plotted versus (*S*)-PhPr concentrations. Drug-induced current responses were specific to GFP+ cells and displayed a dose-dependency (n = 4 cells for each group); two-way ANOVA, group effect: $F_{1, 6}$ = 42.10, p < 0.001, partial η^2 = 0.88, concentration effect: $F_{2, 12}$ = 9.18, p = 0.004, partial η^2 = 0.60, interaction: $F_{12, 48}$ = 10.79, p = 0.002, partial η^2 = 0.64; ΔI of the GFP+ cells were significantly greater than those of GFP- cells at all concentrations*, $Fs_{1,6}$ = 9.66, 11.25, and 26.96, ps = 0.021, 0.015, and 0.002, η^2s = 0.03, 0.49, and 0.81, respectively for 1.0, 5.0 and 10.0 (mM) (*S*)-PhPr; Simple-main effect of concentration were significant for GFP+ but not for GFP+ cells, $Fs_{2, 12}$ = 19.93 and 0.03, p < 0.001 and p = 0.968, η^2s = 0.77 and 0.01, respectively. Data are presented as mean with accompanying error bar (SEM) and individual data points superimposed. Inset shows typical current traces from GFP+ (green) and GFP- cells (gray) to 10 mM (*S*)-PhPr.

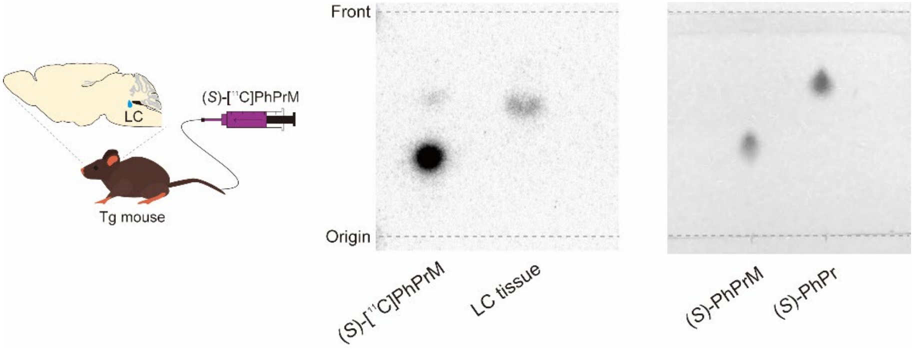

Supplementary Figure 5: TLC analysis of the tissue sample dissected from the LC area of the Tg mouse brain after (*S*)-[^{11}C]PhPrM treatment.

The Tg mouse was intravenously injected with (*S*)-[^{11}C]PhPrM (42 MBq/0.1 ml), and its brain was removed 30 min later to prepare tissue suspension of the LC region for the TLC analysis. The sample and (*S*)-[^{11}C]PhPrM were examined using a radio-TLC scanner (left photo), while the standard compounds were visualized using standard methods (UV light, right photo). Retention factor (Rf) values for (*S*)-[^{11}C]PhPrM and the LC tissue suspension were 0.36 and 0.60, respectively, and Rf values for (*S*)-PhPrM and (*S*)-PhPr were 0.38 and 0.64, respectively. The data indicate that the value obtained from the tissue suspension was similar to that from the standard of (*S*)-PhPr, suggesting the conversion of (*S*)-[^{11}C]PhPrM in the Tg mouse brain.

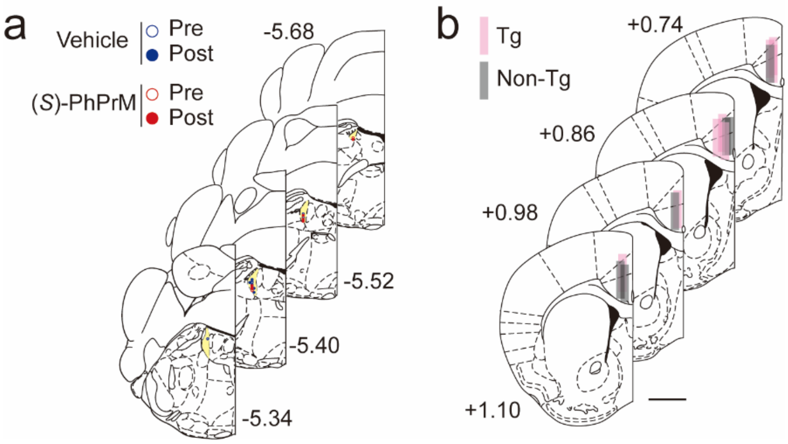

Supplementary Figure 6: Placement sites of glass electrodes for *in vivo* electrophysiology and dialysis probes for *in vivo* microdialysis analysis in Figure 2.

a Site mapping of the tips of glass electrodes in the LC of the Tg mice (for Fig, 2c, d). **b** Site mapping of the dialysis probes in the ACC of the Tg and non-Tg mice (for Fig. 2e). The anteroposterior coordinates (mm) are shown. Scale bar: 1 mm.

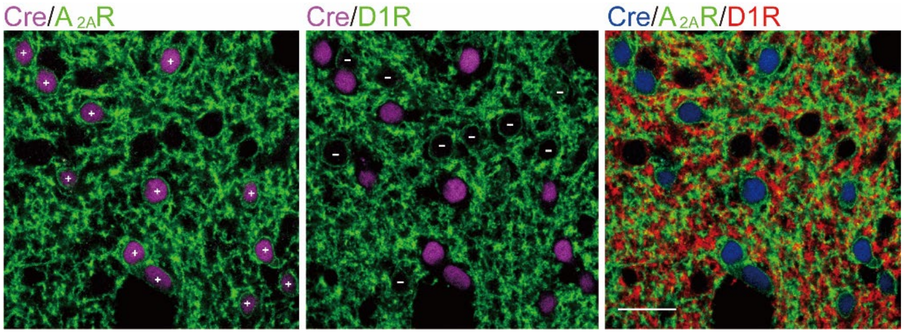

Supplementary Figure 7: Specificity of the Cre recombinase expression in iSPNs of the striatum of the *Drd2*-Cre rats.

Triple immunohistochemistry of the section through the striatum of the *Drd2*-Cre rats for Cre, $A_{2A}R$, and D1R established that the Cre transgene was highly and specifically expressed in iSPNs ($A_{2A}R$-positive), but was absent in dSPNs (D1R-positive). + Cre-positive cell, - Cre-negative cell. Scale bar: 25 μm.

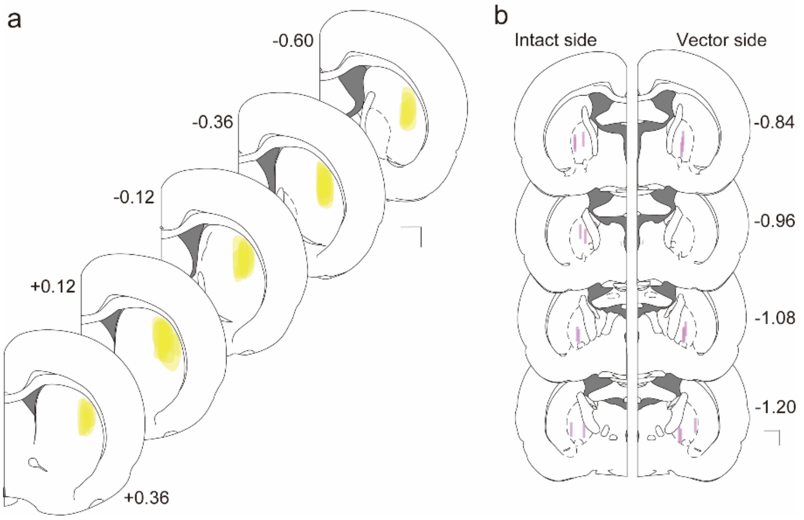

Supplementary Figure 8: Expression patterns of transgenes and placement sites of microdialysis probes.

a Range of IR84a/IR8a expression in the dorsal striatum of the *Dr2d*-Cre rats (for Fig. 4B). **b** Site mapping of the dialysis probes in the GPe of the *Dr2d*-Cre rats (for Fig. 4c, d). The anteroposterior coordinates (mm) are shown. Scale bar: 1 mm.

Supplementary Table 1: Count of various types of immuno-positive neurons in the viral vector-treated striatum of the *Drd2*-Cre rats.

	Section	
Cell classification	Incubated w/ anti- A_{2A}R-antibody	Incubated w/ anti-D1R-antibody
GFP (IR84a)$^+$	65.50 ± 4.94	65.00 ± 5.80
IR8a$^+$	71.00 ± 4.34	68.50 ± 5.55
GPF$^+$ & IR8a$^+$	63.00 ± 3.58	62.25 ± 6.12
$A_{2A}R^+$	68.25 ± 5.22	NA
D1R$^+$	NA	52.50 ± 3.77
GFP$^+$ & $A_{2A}R^+$	51.50 ± 4.29	NA
GFP$^+$ & D1R$^+$	NA	1.25 ± 0.48
IR8a$^+$ & $A_{2A}R^+$	55.25 ± 4.52	NA
IR8a$^+$ & D1R$^+$	NA	1.75 ± 0.48
GFP$^+$ & IR8a$^+$ & $A_{2A}R^+$	50.50 ± 4.17	NA
GFP$^+$ & IR8a$^+$ & D1R$^+$	NA	1.25 ± 0.48
