# Supplementary material for: Chemogenetic activation of mammalian brain neurons expressing insect Ionotropic Receptors by systemic ligand precursor administration
Source: Commun Biol. 2024 May 7;7:547. doi: 10.1038/s42003-024-06223-4 (PMC11076466; doi:10.1038/s42003-024-06223-4)
Supplement: Supplementary file 3 — Reporting-summary [file 42003_2024_6223_MOESM3_ESM.pdf]

nature portfolio

Corresponding author(s): Kazuto Kobayashi

Last updated by author(s): Apr 12, 2024

Reporting Summary

Nature Portfolio wishes to improve the reproducibility of the work that we publish. This form provides structure for consistency and transparency in reporting. For further information on Nature Portfolio policies, see our Editorial Policies and the Editorial Policy Checklist.

Statistics

For all statistical analyses, confirm that the following items are present in the figure legend, table legend, main text, or Methods section.

n/a	Confirmed	
☐	☒	The exact sample size (*n*) for each experimental group/condition, given as a discrete number and unit of measurement
☐	☒	A statement on whether measurements were taken from distinct samples or whether the same sample was measured repeatedly
☐	☒	The statistical test(s) used AND whether they are one- or two-sided<br>*Only common tests should be described solely by name; describe more complex techniques in the Methods section.*
☒	☐	A description of all covariates tested
☐	☒	A description of any assumptions or corrections, such as tests of normality and adjustment for multiple comparisons
☐	☒	A full description of the statistical parameters including central tendency (e.g. means) or other basic estimates (e.g. regression coefficient) AND variation (e.g. standard deviation) or associated estimates of uncertainty (e.g. confidence intervals)
☐	☒	For null hypothesis testing, the test statistic (e.g. F, t, r) with confidence intervals, effect sizes, degrees of freedom and P value noted<br>*Give P values as exact values whenever suitable.*
☒	☐	For Bayesian analysis, information on the choice of priors and Markov chain Monte Carlo settings
☒	☐	For hierarchical and complex designs, identification of the appropriate level for tests and full reporting of outcomes
☐	☒	Estimates of effect sizes (e.g. Cohen's *d*, Pearson's *r*), indicating how they were calculated

Our web collection on statistics for biologists contains articles on many of the points above.

Software and code

Policy information about availability of computer code

Data collection	The rotation information of animals was transferred to a personal computer via an interface (USB-6501, National Instruments) and recorded using a Matlab simulink-based application (Matlab R2019b, Mathworks).
Data analysis	All statistical analyses were two-tailed and conducted using SPSS ver. 25 (IBM). The reliability of the results was assessed against a type I error (α) of 0.05.

For manuscripts utilizing custom algorithms or software that are central to the research but not yet described in published literature, software must be made available to editors and reviewers. We strongly encourage code deposition in a community repository (e.g. GitHub). See the Nature Portfolio guidelines for submitting code & software for further information.

Data

Policy information about availability of data

All manuscripts must include a data availability statement. This statement should provide the following information, where applicable:

- Accession codes, unique identifiers, or web links for publicly available datasets
- A description of any restrictions on data availability
- For clinical datasets or third party data, please ensure that the statement adheres to our policy

The datasets generated and analyzed in this study are deposited on Mendeley data. DOI: 10.17632/b47j739jb7.1
https://data.mendeley.com/datasets/b47j739jb7/1

March 2021

Human research participants

Policy information about studies involving human research participants and Sex and Gender in Research.

Reporting on sex and gender	n/a
Population characteristics	n/a
Recruitment	n/a
Ethics oversight	n/a

Note that full information on the approval of the study protocol must also be provided in the manuscript.

Field-specific reporting

Please select the one below that is the best fit for your research. If you are not sure, read the appropriate sections before making your selection.

☒ Life sciences ☐ Behavioural & social sciences ☐ Ecological, evolutionary & environmental sciences

For a reference copy of the document with all sections, see nature.com/documents/nr-reporting-summary-flat.pdf

Life sciences study design

All studies must disclose on these points even when the disclosure is negative.

Sample size	Although no statistical methods were used to predetermine the sample size for each measure, we employed similar sample sizes to those reported in previous publications from our labs, which have been generally accepted in the field.
Data exclusions	n/a
Replication	n/a
Randomization	The assignment of animals to the experimental conditions was random.
Blinding	n/a

Reporting for specific materials, systems and methods

We require information from authors about some types of materials, experimental systems and methods used in many studies. Here, indicate whether each material, system or method listed is relevant to your study. If you are not sure if a list item applies to your research, read the appropriate section before selecting a response.

Materials & experimental systems

n/a	Involved in the study	
☐	☒	Antibodies
☐	☒	Eukaryotic cell lines
☒	☐	Palaeontology and archaeology
☐	☒	Animals and other organisms
☒	☐	Clinical data
☒	☐	Dual use research of concern

Methods

n/a	Involved in the study	
☒	☐	ChIP-seq
☒	☐	Flow cytometry
☒	☐	MRI-based neuroimaging

Antibodies

Antibodies used	Primary antibodies for Cre (mouse, 1:200; MAB3120, Sigma-Aldrich), A2AR (goat, 1:200; A2A-Go-Af700, Frontier Institute), and D1R (guinea pig, 1:200; D1R-GP-Af500, Frontier Institute) (Supplementary Fig. 7), or GFP (chicken, 1:1,000; ab13970, Abcam), IR8a (guinea pig, 1:1,000; Abuin et al., 2011), and A2AR, or GFP, IR8a, and D1R (goat, 1:200, D1R-Go-Af1000, Frontier Institute) (Fig. 3a), or GFP and IR8a (Supplementary Fig. 8a) were used. Alexa Fluor 647-conjugated donkey anti-mouse antibody (A-31571, Thermo Fisher Scientific), Alexa Fluor 488-conjugated donkey anti-goat antibody (A-11055, Thermo Fisher Scientific), Cy3-conjugated donkey anti-guinea pig antibody (706-165-148, Jackson ImmunoResearch Laboratories), Alexa Fluor 488-conjugated donkey anti-chicken antibody (703-545-155, Jackson ImmunoResearch Laboratories), Alexa Fluor 647-conjugated donkey anti-goat antibody (A-21447, Thermo

	Fisher Scientific) were used as species-specific secondary antibodies for detecting Cre, A2AR, D1R, GFP, and A2AR, respectively (all dilutions were 1:200).
Validation	All primary antibodies used in this study are validated by the manufacturers and also validated by following previous reports:<br>anti-IR8a: Abuin et al., Neuron, 2011<br>anti-D1R, anti-A2AR: Nonomura et al., Neuron, 2018

Eukaryotic cell lines

Policy information about cell lines and Sex and Gender in Research

Cell line source(s)	HEK293T and NG108-15 were obtained from ATCC (CRL-11268 and HB12317, respectively).
Authentication	Cell lines were not authenticated after purchase.
Mycoplasma contamination	Cell lines were not tested for mycoplasma contamination but no indication of contamination was observed.
Commonly misidentified lines<br>(See ICLAC register)	No commonly misidentified cell lines were used.

Animals and other research organisms

Policy information about studies involving animals; ARRIVE guidelines recommended for reporting animal research, and Sex and Gender in Research

Laboratory animals	Tg mice including B6.Cg-Tg(TH-GFP-IR84a/IR8a)2-1Koba and B6.B6D2-Tg(Th-EGFP)21-31Koba (Riken BioResource Research Center) were bred with wild-type C57BL/6J mice (CLEA Japan) to produce the heterozygous and wild-type offspring. LE-Tg(Drd2-cre)490-9Koba Tg rats (The National BioResource Project for the Rat in Japan) were mated with wild-type Long Evans rats (Charles River Laboratories) to obtain the heterozygous and wild-type offspring.
Wild animals	n/a
Reporting on sex	Male rats were used in the behavioral and biochemical experiment. In all other experiments, both males and females were used.
Field-collected samples	n/a
Ethics oversight	All procedures were approved by the Fukushima Medical University Institutional Animal Care and Use Committee and RIKEN Biosystems Dynamics Research.

Note that full information on the approval of the study protocol must also be provided in the manuscript.
