# Supplementary material for: Chemogenetic activation of mammalian brain neurons expressing insect Ionotropic Receptors by systemic ligand precursor administration
Source: Commun Biol. 2024 May 7;7:547. doi: 10.1038/s42003-024-06223-4 (PMC11076466; doi:10.1038/s42003-024-06223-4)
Supplement: Supplementary file 1 — Peer Review File [file 42003_2024_6223_MOESM1_ESM.pdf]

nature portfolio

Peer Review File

Chemogenetic activation of mammalian brain neurons expressing insect Ionotropic Receptors by systemic ligand precursor administration

Open Access This file is licensed under a Creative Commons Attribution 4.0 International License, which permits use, sharing, adaptation, distribution and reproduction in any medium or format, as long as you give appropriate credit to the original author(s) and the source, provide a link to the Creative Commons license, and indicate if changes were made. In the cases where the authors are anonymous, such as is the case for the reports of anonymous peer reviewers, author attribution should be to 'Anonymous Referee' followed by a clear attribution to the source work. The images or other third party material in this file are included in the article's Creative Commons license, unless indicated otherwise in a credit line to the material. If material is not included in the article's Creative Commons license and your intended use is not permitted by statutory regulation or exceeds the permitted use, you will need to obtain permission directly from the copyright holder. To view a copy of this license, visit http://creativecommons.org/licenses/by/4.0/.

Reviewers' comments:

Reviewer #1 (Remarks to the Author):

In this manuscript by Iguchi and colleagues, the authors describe the use of a blood-brain-barrier permeable ligand for a recently characterized chemogenetic construct based on an insect ionic channel sensitive to phenylacetic acid. The work is globally of high quality and convincing and demonstrates that the new methyl ester form of phenylacetic acid is able to enter the brain after peripheral administration and induce activation of the insect ionotropic receptor expressed either in locus coeruleus noradrenergic neurons or in striatal GABAergic neurons, leading to increased firing, increased NE and GABA release and also altered motor behaviors. The manuscript would nonetheless benefit from being revised to take care of the following issues:
1. It would be useful to provide some more context for the work in the abstract. For example, why are new chemogenetic approaches needed? To solve what problems? What is the added value of using ionotropic instead of metabotropic receptors for this? Some of this is addressed in the introduction, but it would be good to give a flavor of this in the abstract as well.
2. The discussion of the paper needs to address more clearly the main limitations of the proposed approach. For example, the approach did not lead to very robust increases in neuronal firing rates in the in vivo experiments. Also, the effects obtained were quite slow. This needs to be discussed.
3. The results never show any dose response relationships obtained with the phenylacetic acid ligand. Why is that? New data should ideally be added to show this, or, at the minimum, this should be discussed.
4. For the experiments of figure 4, with striatal expression of IR, I am puzzled as to why the authors only waited 10 min after the IP administration of PhAcM before administering cocaine? This is much shorter than the time required to activate NE neurons and as such, this suggests that this is not enough time to obtain maximal effects. This needs to be justified.
5. Was the PET imaging experiment performed in a single rat? If not, why is the signal not quantified? This needs to be clarified in the text.
6. In the discussion, the authors need to more clearly explain what biological questions, if any, they wanted to tackle by expressing IR in the locus coeruleus and striatal neurons. The discussion includes a lengthy discussion about striatal functions, but this is not well linked to anything addressed in the introduction.
7. On page 22 of the discussion, when the authors compare their approach to more standard chemogenetic approaches, it would be good to provide an example of an experiment that could be performed with IRNA but that could not be performed with more standard chemogenetic approaches.
8. In the histology part of the methods section, the authors refer to the use of a vibratome. The word “vibrating blade microtome” should be used instead because “Vibratome” is a trademark.
9. The quality of the English needs to be improved throughout the manuscript.

Reviewer #2 (Remarks to the Author):

Iguchi et al. reported a new method for chemogenetic activation of neurons in the brain through

peripheral administration of the ligand. They utilized insect ionotropic receptors (IRs) composed of IR84a and IR8a subunits. The group has previously reported chemogentic activation of catecholamine-containing neurons by intracranial microinjection of phenylacetic acid (PhAc), a selective ligand for the IRs. However, PhAc has the difficulty penetrating the blood-brain barrier. In this paper, the authors developed methyl ester of PhAc (PhAcM) and (S)-PhPrM for peripheral administration, which had BBB permeability. Notably, these compounds were converted to the active species (PhAc and PhPr) by esterase activities in the brain. They successfully applied this strategy for the selective activation of target neurons in the brains of Cre transgenic rats infected with viral vectors. Importantly, they visualized the binding of the ligand in vivo using PET imaging. Overall, the manuscript is well written, and these new techniques will be useful for chemogenetic control of nerve activity in animals. However, this reviewer raises several points to improve this paper. These need to be addressed to improve this manuscript.

1. In Supplementary Fig. 1b, high concentrations (> 300 microM) of PhAc are required for elevation of extracellular NE in the Tg mice. To investigate the side effects, the effect of PhAc to wild-type mice should be examined over the same concentration range.

2. The authors claim that PhAcM and PhPrM are converted to PhAc and PhPr by esterase activities in the brain. However, any references have not been cited in the current manuscript. Add the appropriate references. In addition, this reviewer is concerned that esterases are also expressed in blood and degrade PhAcM and PhPrM. The authors need to discuss it.

3. According to the previous paper (ref. 25), the EC50 values of PhAc for IR84a and IR8a range from 0.1 to 1.0 mM. This range is consistent with the current data shown in Supplementary Fig. 1 where PhAc was administered intracranially. In contrast, it would be generally difficult to add the same range of the PhAc precursor, PhAcM to the brain by tail vein injection. However, in this paper, the authors successfully activated the target neuron by peripheral administration of PhAc. The authors should discuss the discrepancy.

4. The authors have newly developed the (S)-PhPr as a ligand for IR84a/IR8a. However, pharmacological evaluation is insufficient. The authors need to examine pharmacological assays such as the concentration dependency of (S)-PhPr on IR84a/IR8a activation.

5. Page 15, line 8; the authors describe the concentration of PhAc using molarity rather than %.

Point-by-point responses to the reviewers' comments

◆**Reviewer #1**

In this manuscript by Iguchi and colleagues, the authors describe the use of a blood-brain-barrier permeable ligand for a recently characterized chemogenetic construct based on an insect ionic channel sensitive to phenylacetic acid. The work is globally of high quality and convincing and demonstrates that the new methyl ester form of phenylacetic acid is able to enter the brain after peripheral administration and induce activation of the insect ionotropic receptor expressed either in locus coeruleus noradrenergic neurons or in striatal GABAergic neurons, leading to increased firing, increased NE and GABA release and also altered motor behaviors. The manuscript would nonetheless benefit from being revised to take care of the following issues:

Response:

We thank you for your kind and positive comments, which have helped us to improve our manuscript. We have responded to each comment, point by point, below. All revised text is shown in red font in the new manuscript.

Comment 1:

It would be useful to provide some more context for the work in the abstract. For example, why are new chemogenetic approaches needed? To solve what problems? What is the added value of using ionotropic instead of metabotropic receptors for this? Some of this is addressed in the introduction, but it would be good to give a flavor of this in the abstract as well.

Response:

According to your advice, we have revised the abstract and added the following sentences at the beginning, which simply describe the importance of the development of the LGIC-based chemogenetic tool.

"Chemogenetic approaches employing ligand-gated ion channels are advantageous regarding manipulation of target neuronal population functions independently of the intrinsic second messenger pathways. Among them, Ionotropic Receptor (IR)-mediated neuronal activation (IRNA) allows..." (page 4)

Comment 2:

The discussion of the paper needs to address more clearly the main limitations of the proposed approach. For example, the approach did not lead to very robust increases in neuronal firing rates in the in vivo experiments. Also, the effects obtained were quite slow. This needs to be discussed.

Response:
The increase in firing rate of the IR84a/IR8a expressing LC-NE neurons following peripheral administration of PhAcM observed in the present study (Fig. 1b) was milder than the effect on the firing rate of the same neuronal population by the intracranial microinjection of PhAc in our previous reports (Fukabori et al., *J. Neurosci.*, 2020). The milder effect of PhAcM administration may be explained by lower concentrations of PhAc in the brain region provided from the ligand precursor. Thus, it remains to be clarified the extent of conversion from the ligand precursor to the active ligand in the target brain regions. We have discussed the limitation of the peripheral pro-drug delivery system in the revised manuscript (page 21, line 18–page 22, line 7).

In addition, the time course of the physiological effects also differed between ligand precursors, showing a delayed onset for (*S*)-PhPrM compared to PhAcM. The difference in time course may be attributable to the complex metabolic processes, such as the precursor translocation into the brain and active form conversion. In particular, conformational interference in the active site of esterase is known to reduce the efficiency of the hydrolysis (Imai et al., *Drug Metab. Dispos.*, 2006), and the changed hydrolysis may affect the time course of (*S*)-PhPr bioavailability in the brain regions. We have described this limitation in the discussion section (page 21, lines 5–18).

Comment 3:
The results never show any dose response relationships obtained with the phenylacetic acid ligand. Why is that? New data should ideally be added to show this, or, at the minimum, this should be discussed.

Response:
In our previous report (Fukabori et al., *J. Neurosci.*, 2020), we reported a dose-dependent response of the IR84a/IR8a-expressing cells to PhAc using a patch clamp experiment with cultured cells and *in vivo* microdialysis with the Tg mice. In the present study, we also presented *in vivo* micro (reverse) dialysis data showing a dose-dependence of PhAc stimulation on norepinephrine (NE) release in the terminal region of the noradrenergic system of the Tg mice in Supplementary Figure 1.

Comment 4:
For the experiments of figure 4, with striatal expression of IR, I am puzzled as to why the authors only waited 10 min after the IP administration of PhAcM before administering cocaine? This is much shorter than the time required to activate NE neurons and as such, this suggests that this is not enough time to obtain maximal effects. This needs to be justified.

Response:

Our *in vivo* extracellular recording of the NE neurons in Tg mice showed that a significant increase in firing rate occurred approximately 10 min after peripheral PhAcM administration. Based on these data, we set the interval between the administrations of PhAcM and cocaine in rats, and started monitoring behavior immediately after the cocaine was administered.

We have added the following explanation on why we chose to set the interval to 10 min between PhAcM and cocaine administration in the corresponding section (page 16, lines 13–17).

"Rats were injected intraperitoneally (i.p.) with cocaine (15 mg/kg), and their behavior was monitored 10 min after administration of PhAcM i.p. (10 mg/kg), since our *in vivo* electrophysiological experiments with Tg mice showed that peripheral PhAcM administration resulted in significant elevation of the target neurons' firing rate after 10 min (Fig. 1b)."

Comment 5:
Was the PET imaging experiment performed in a single rat? If not, why is the signal not quantified? This needs to be clarified in the text.

Response:
We performed PET imaging in three rats, quantified PET data, and found a significant increase in the labeled ligand binding in the vector-treated side of the striatum. We have therefore added the descriptions to the Results (page 19, lines 4–7) and Methods (page 42, lines 3–9). A new figure panel (Fig. 5c, see below) and its figure legend (page 70, lines 8–11) were also included.

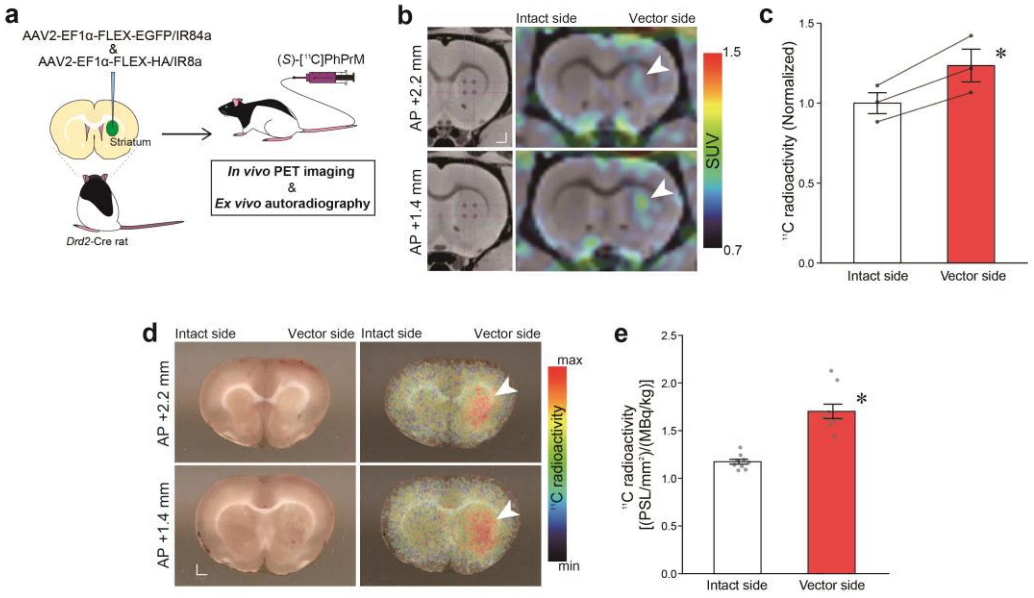

Comment 6:
In the discussion, the authors need to more clearly explain what biological questions, if any, they wanted to tackle by expressing IR in the locus coeruleus and striatal neurons. The discussion includes a lengthy discussion about striatal functions, but this is not well linked to anything addressed in the introduction.

Response:
In our previous report (Fukabori et al., *J. Neurosci.*, 2020), we tried to elucidate the mechanism of noradrenergic circuit that regulates the retrieval of emotional memory using the IRNA technology. On the other hand, in the present study we aimed to improve the utility of the technology by developing a pro-drug system in which the ligand precursor is peripherally administered and converted to the mature ligand. Indeed, we successfully achieved the pro-drug strategy that activates target neurons in the brain, resulting in increased neurotransmitter release and behavioral changes. We believe that our systemic extension of the IRNA technology will contribute to future studies that analyze a variety of brain functions in detail.

Comment 7:
On page 22 of the discussion, when the authors compare their approach to more standard chemogenetic approaches, it would be good to provide an example of an experiment that could be performed with IRNA but that could not be performed with more standard chemogenetic approaches.

Response:
Accurate benchmark comparisons between our (systemic) IRNA technology and existing approaches such as DREADDs are not easy, because these approaches have been applied within separate experimental paradigms. A systemic comparison of their effects within a single paradigm targeting the same neuronal population would be an important direction for future research. We have revised the manuscript to clearly state this point in the Discussion (page 23, line 17–page 24, line 3).

"Accurate benchmark comparisons between the existing chemogenetic systems and the IRNA technology are difficult since each of the approaches so far has been used in its own distinct experimental paradigm, and systematic comparison of the effects in a similar paradigm targeting the same neuronal population is a future challenge."

Comment 8-9:
In the histology part of the methods section, the authors refer to the use of a vibratome. The word "vibrating blade microtome" should be used instead because "Vibratome" is a trademark.

The quality of the English needs to be improved throughout the manuscript.

Response:
Thank you for pointing this out. We have now made the necessary correction (page 32, lines 4–5).

The main text and Supplementary Information files have been proofread by an English native speaker.

◆Reviewer #2

Iguchi et al. reported a new method for chemogenetic activation of neurons in the brain through peripheral administration of the ligand. They utilized insect ionotropic receptors (IRs) composed of IR84a and IR8a subunits. The group has previously reported chemogentic activation of catecholamine-containing neurons by intracranial microinjection of phenylacetic acid (PhAc), a selective ligand for the IRs. However, PhAc has the difficulty penetrating the blood-brain barrier. In this paper, the authors developed methyl ester of PhAc (PhAcM) and (S)-PhPrM for peripheral administration, which had BBB permeability. Notably, these compounds were converted to the active species (PhAc and PhPr) by esterase activities in the brain. They successfully applied this strategy for the selective activation of target neurons in the brains of Cre transgenic rats infected with viral vectors. Importantly, they visualized the binding of the ligand in vivo using PET imaging. Overall, the manuscript is well written, and these new techniques will be useful for chemogenetic control of nerve activity in animals. However, this reviewer raises several points to improve this paper. These need to be addressed to improve this manuscript.

Response:
We thank you for providing so much useful feedback, which has helped us to improve our manuscript. We have responded to each comment, point by point, below. All revised text is in red font in the new manuscript.

Comment 1:
In Supplementary Fig. 1b, high concentrations (> 300 microM) of PhAc are required for elevation of extracellular NE in the Tg mice. To investigate the side effects, the effect of PhAc to wild-type mice should be examined over the same concentration range.

Response:
As the reviewer suggested, we tested whether 300–3,000 μM PhAc had the effect on NE release in the wild type mice. There were no significant changes in NE release after treatment with PhAc of these concentrations. We added the data to Supplementary

Information (Supplementary Fig. 1b, see below) and revised the relevant parts of the main text (page 9, lines 11–12).

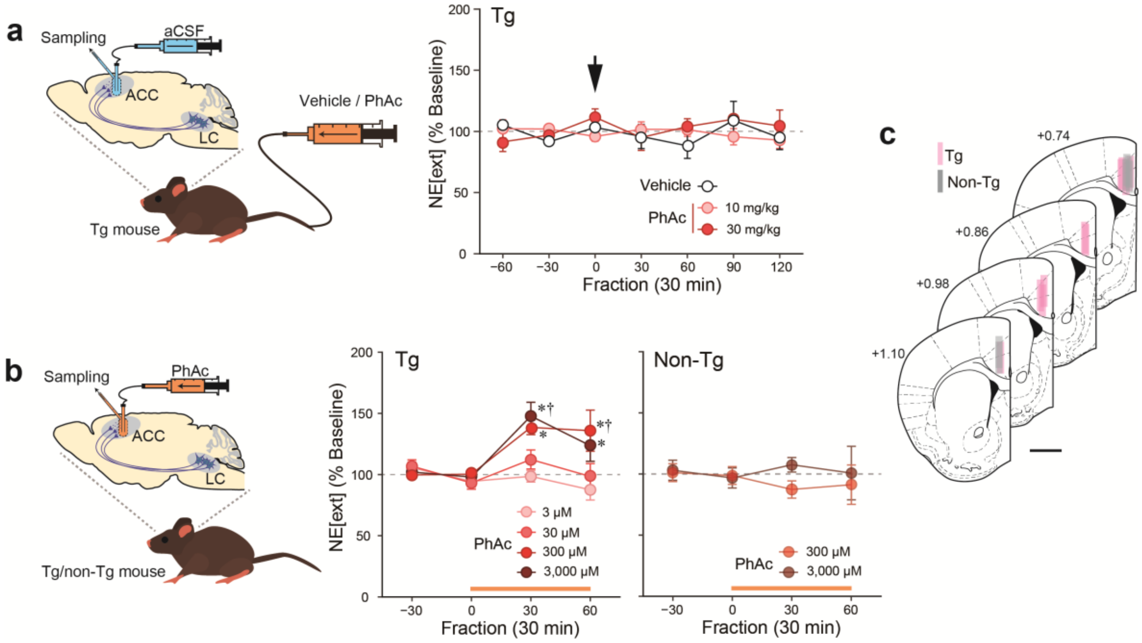

Comment 2:

The authors claim that PhAcM and PhPrM are converted to PhAc and PhPr by esterase activities in the brain. However, any references have not been cited in the current manuscript. Add the appropriate references. In addition, this reviewer is concerned that esterases are also expressed in blood and degrade PhAcM and PhPrM. The authors need to discuss it.

Response:

Because previous studies already cited in the manuscript (refs. 30-32) showed a conversion from a methyl ester form to the carboxylic acid form by esterase activities in addition to a higher BBB permeability, we revised the explanation of the relevant references in the text (page 9, lines 16–17).

Because esterase activities in the blood have previously been reported, as you have pointed out, we addressed the possibility that the ligand precursor may be converted not only in the brain but also in the blood (page 20, lines 13–14), citing the following two newly added references that reported esterase activities in the brain (ref. 41) as well as in the blood (ref. 42).

"It should be noted that the conversion would also occur in blood, in which esterase activities have previously been reported[42]."

41. Eto, Y. & Suzuki, K. Cholesterol ester metabolism in rat brain: A cholesterol ester hydrolase specifically localized in the myelin sheath. *J. Biol. Chem.* **25**, 1986-1991, doi:10.1016/S0021-9258(19)44176-8 (1973).
42. Choudhury, S. R. Sensitivity of carboxylesterases of rat serum to diethyl-p-nitrophenyl phosphate. *Enzyme* **21**, 349-356, doi:10.1159/000458878 (1976).

Comment 3:
According to the previous paper (ref. 25), the EC50 values of PhAc for IR84a and IR8a range from 0.1 to 1.0 mM. This range is consistent with the current data shown in Supplementary Fig. 1 where PhAc was administered intracranially. In contrast, it would be generally difficult to add the same range of the PhAc precursor, PhAcM to the brain by tail vein injection. However, in this paper, the authors successfully activated the target neuron by peripheral administration of PhAc. The authors should discuss the discrepancy.

Response:
Peripheral administration of PhAcM resulted in a significant increase in firing rate of the IR84a/IR8a expressing LC-NE neurons in the present study, but the increase was milder than the effect on the firing rate of the same neuronal population by the intracranial microinjection of PhAc as previously reported. The milder effect of PhAcM administration may be explained by lower concentrations of PhAc in the brain region provided from the ligand precursor. In the reverse dialysis experiment in mice, we obtained significant results on the effects of noradrenergic stimulation when PhAc was perfused at 300–3,000 µM in the dialysis probe. On the other hand, we calculated the rate of PhAcM transfer into the brain based on the PET data, and the rate was approximately 0.2%. Under this condition, when the translocation of the ligand precursor and endogenous esterase activity are hypothesized to be uniform in the whole brain, the concentration of PhAc in the brain was calculated to be about 30 µM after administration of 10 mg/kg of peripheral PhAcM. This concentration is comparable to approximately 10% of that used for intracranial injection or microdialysis perfusion. However, there is the possibility that the efficiency of the conversion may be different among the brain regions, and that peripheral administration of PhAcM may result in higher concentrations of ligand accumulated in the target brain regions. Therefore, we need to determine the concentration of PhAc in specific brain regions in the future. We have summarized the possibility to explain the difference in the effects between the peripheral and intracranial injections in the discussion section (page 21, line 18–page 22, line 7).

Comment 4:
The authors have newly developed the (S)-PhPr as a ligand for IR84a/IR8a. However, pharmacological evaluation is insufficient. The authors need to examine pharmacological assays such as the concentration dependency of (S)-PhPr on IR84a/IR8a activation.

Response:
We expressed the IR84a/IR8a complex in NG108-15 cell line to examine the responses to different concentrations of (*S*)-PhPr. The IR84a/IR8a-expressing cells showed a dose-dependent current response to 1 to 10 mM (*S*)-PhPr.

We added the data as Supplementary Figure 4 (page 7 in Supplementary Information, see below) with the relevant methodology used in the main text (for viral vector preparation, page 28 lines 3–5; for cell preparation, page 30, lines 6–9; for patch clamp experiment, page 35, lines 7–17; and for three newly added references, page 56).

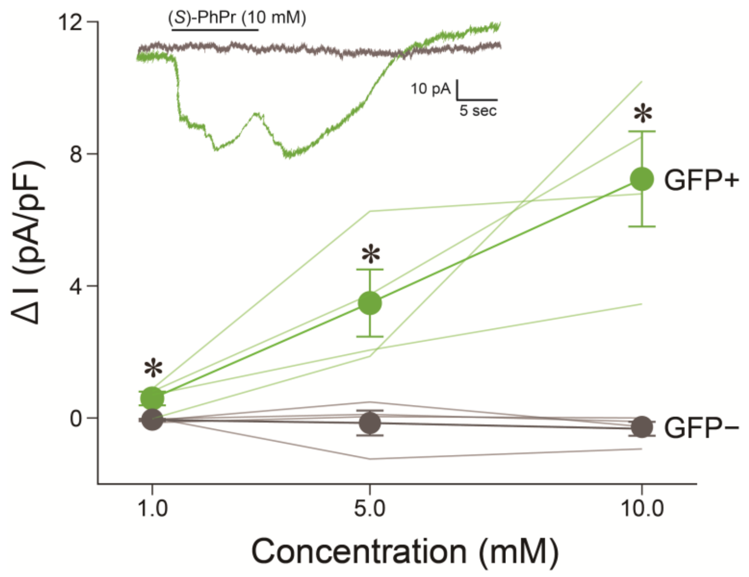

Comment 5:
Page 15, line 8; the authors describe the concentration of PhAc using molarity rather than %.

Response:
We have now added the concentration of PhAc in molarity as well as % w/v (page 15, line 12).

REVIEWERS' COMMENTS:

Reviewer #1 (Remarks to the Author):

The authors' responses and manuscript revisions adequately addressed my previous concerns.

Reviewer #2 (Remarks to the Author):

The authors have done a great job addressing to my comments.
All concerns for me were solved in the revised manuscript.
